# From Integrated Care to Learning Systems

**DOI:** 10.3390/healthcare14121612

**Published:** 2026-06-08

**Authors:** Aristeidis Tsitiridis, Konstantinos Perakis, Athos Antoniades, George Manias

**Affiliations:** 1 Face Recognition & Artificial Vision, Escuela Técnica Superior de Ingeniería Informática (ETSII), Universidad Rey Juan Carlos, Calle Tulipan S/N, 28933 Mostoles, Madrid, Spain; 2 Department of Informatics and Systems Engineering, Defence Academy of the UK, Cranfield University, Shrivenham, Swindon SN6 8LA, UK; 3 Ubitech Ltd., 15231 Chalandri, Greece; kperakis@ubitech.eu; 4 Stremble Ventures Ltd., 4042 Limassol, Cyprus; athos.antoniades@stremble.com; 5 Department of Digital Systems, University of Piraeus, Karaoli & Dimitriou 80, 18534 Piraeus, Greece; gmanias@unipi.gr

**Keywords:** integrated care, integrated care models, artificial intelligence, learning health systems, stewarded learning, data governance, multimodal analytics, large language models, EU AI Act, FUTURE-AI, PRISMA-ScR, scoping review, global health, healthy ageing

## Abstract

Integrated care is increasingly shaped by digital infrastructures, data governance, and AI-enabled analytics, yet the relevant literature remains fragmented across health-services research, digital health, and machine learning. This article reports a scoping review, conducted in line with PRISMA-ScR guidance, that maps how integrated care models have evolved conceptually, what digital and AI-enabled infrastructures support them, how their clinical, economic, and equity impacts can be evaluated, and what current implementations imply for sustainable scaling. We searched PubMed, Scopus, Semantic Scholar, and Crossref (retrieval date 31 October 2025; forward screening to 31 March 2026) and added grey literature from named policy bodies. The searches identified 15,189 records, reducing to 11,789 after intra- and cross-source deduplication and grey-literature integration; 620 full texts were assessed and 192 were included in the synthesis. Four domains were synthesised: conceptual foundations of integrated care, AI and multimodal analytics, implementation barriers, and digital-governance foundations. We chart the field using a Type I–V maturity scheme (disease, cohort, whole-system, digital-integrated, learning), benchmarked against the Rainbow, MacColl, EMRAM/AMAM, and NHS ICS models. Most deployments cluster at digitally integrated but only weakly adaptive Type IV; recurrent failure modes—temporal blind spots, maintenance debt, semantic drift, and governance gaps—block progression to Type V, and high-profile clinical-AI failures illustrate the cost of attempting Type V analytics on Type IV-or-worse infrastructure. A walk through nine world regions maps each to its current Type I–V position and shows that organisational and payment integration—not digital sophistication alone—is currently the dominant driver of progress. The COMFORTage Integrated Care Model Library is positioned as a workflow of AI agents orchestrating predictive, preventive, and personalised care across the integrated-care lifecycle rather than as a single federated-learning programme. The review positions AI-enabled integrated care less as a finished model than as an emerging design space requiring longitudinal data assets, stewarded model lifecycles, accountable governance, and outcome-based contracting for clinically useful, equitable, and trustworthy learning systems.

## 1. Introduction

Health systems worldwide are facing a demographic and epidemiological inflection point. By 2050, one in six people globally will be aged 65 or older [1], and in the European Union, this proportion will approach one-third [2]. Life expectancy continues to rise, but so too does the prevalence of multimorbidity, frailty, and cognitive decline [3]. Non-communicable diseases now account for nearly three-quarters of global mortality [4], and the OECD projects a shortfall exceeding ten million long-term-care workers by 2040, with informal caregivers absorbing more responsibility without training or compensation [5,6]. Care demand, workforce pressure, and social dependency now interact across the sectoral boundaries that integrated care is meant to bridge [7], and traditional disease-centred services, organised around specialities and institutions rather than individuals, are proving inadequate for the long-term coordination and prevention these populations require [8,9,10].

Empirically, care fragmentation is associated with higher utilisation, more avoidable hospitalisations, and medication safety risks, especially for older adults with multimorbidity [11,12,13,14]. Cross-regional analyses suggest a sizeable share of variation in fragmentation is unrelated to clinical need, implying remediable system-design factors [15]. Continuity (seeing the right people at the right time) and coordination (those people working from the same plan and data) are both necessary, and both suffer when digital and organisational seams are misaligned [16]. The result is duplication, waste, and inequitable outcomes that fall hardest on older adults, migrants, and rural populations.

In response, major jurisdictions have formalised integration as both moral and economic necessity. Three anchor frameworks directly shape learning systems in this space: WHO’s Integrated, People-Centred Health Services (IPCHS), the UN Decade of Healthy Ageing, and the European Health Data Space (EHDS) [17,18,19]. Additional policy instruments (the EU Green Paper on Ageing, the European Care Strategy, national digital-health legislation, and regional standards) are summarised in Supplementary Material. Yet two decades of policy notwithstanding, most deployed Integrated Care Models (ICMs) plateau at *digital integration* (dashboards, data-sharing, and one-off analytics, i.e., Type IV) rather than embedding adaptive learning into the governance layer itself (Type V). The IV/V gap is the central tension this review addresses: technology and policy have made digital integration achievable; what remains is the leap to systems that learn.

The conceptual contribution offered here is the Type I–V scheme, used as an analytical framework for charting (Section 4). Types I–III span familiar service-design progression from disease management to whole-system integration; Type IV adds digital scaffolding and predictive analytics; and Type V adds a stewarded learning loop with longitudinal multimodal capture of *patient outcome trajectories*, by which we mean person-level outcome paths that unfold across years (frailty progression, cognitive decline, care-dependency transitions and the response to interventions over time, rather than single visits or episodes), federated AI, and outcome-based contracts. Throughout the rest of the paper, “trajectories” is used in this specific sense unless otherwise qualified. We treat the scheme as a vocabulary for the IV/V distinction, not as a psychometrically validated instrument; Section 4.2 aligns it with existing maturity models (Rainbow, MacColl, EMRAM/AMAM, NHS ICS, WHO GSDH).

This review consolidates evidence at the intersection of health-system design, data governance, and digital innovation. It addresses four guiding questions, stated here in the Introduction: (1) how have ICMs evolved conceptually, and what frameworks underpin them? (2) what digital infrastructures and analytical tools enable integration across domains? (3) which evaluation methods best capture clinical, economic, and equity impacts? and (4) what lessons from current implementations across world regions inform sustainable, equitable scaling, including in low- and middle-income settings? The synthesis is deliberately global in scope, drawing implementation evidence from North America, Europe (including pan-European and EU-wide programmes), the United Kingdom, the Middle East, East Asia, South and Southeast Asia, sub-Saharan Africa, Latin America, and Oceania, so that the Type I–V scheme is read against a regionally balanced evidence base rather than a hidden high-income centre of gravity. Throughout, we signpost *evidence* (claims with empirical support) and *aspiration* (claims about a target architecture not yet routinely achieved). The remainder is organised as Methods (Section 2), Search Results (Section 3), Conceptual Foundations (Section 4), AI and Multimodal Analytics (Section 5), Implementation Barriers (Section 6), Digital and Governance Foundations (Section 7), Discussion (Section 8), Future Directions (Section 9), and Conclusions (Section 10).

## 2. Materials and Methods

### 2.1. Review Type and Reporting Framework

This work is a scoping review with an explicit conceptual-synthesis aim. We adopted the framework proposed by Arksey and O’Malley and refined by Levac et al. [20,21], and we report it in line with the Preferred Reporting Items for Systematic Reviews and Meta-Analyses extension for Scoping Reviews (PRISMA-ScR) [22]. We use “scoping review” here in the sense intended by PRISMA-ScR: a transparent and reproducible mapping of a heterogeneous literature, organised to identify anchor frameworks, representative implementations, and emerging technical directions at the intersection of integrated care, digital health, and artificial intelligence (AI). We deliberately did not undertake a systematic review with risk-of-bias appraisal and pooled effect estimation, because the underlying corpus mixes conceptual papers, policy documents, technical methods, implementation reports, and empirical evaluations whose endpoints are not commensurate; and we did not undertake a purely narrative review, because the volume of recent activity in this space (2024–2026 in particular) makes an undocumented selection process indefensible. The PRISMA-ScR checklist is provided as Supplementary Material (Table S1).

### 2.2. Eligibility Criteria

Inclusion and exclusion criteria are summarised in Table 1. They were applied iteratively, in line with PRISMA-ScR guidance, and refined once during piloting after the first 100 title and abstract screens.

### 2.3. Information Sources and Search

We searched four bibliographic databases (PubMed, Scopus, Semantic Scholar, and Crossref) on a single retrieval date (31 October 2025) and complemented them with targeted forward screening for newly indexed 2025–2026 items up to 31 March 2026. Grey literature was retrieved from the WHO, OECD, European Commission, the U.S. Office of the National Coordinator for Health Information Technology, and national digital-health agencies, on the same retrieval window. The project’s existing COMFORTage reference library (*n* = 150) was cross-checked to retain foundational and policy items already known to the team; 27 exact DOI matches plus 18 high-similarity title matches were carried forward.

We organised the searches around three conceptual tiers reflecting the spread of relevant terminology across health-services research, digital health, and AI:

- Tier 1, AI-intensive corpus: machine learning, deep learning, predictive analytics, and multimodal decision support applied to healthcare and chronic-care delivery.
- Tier 2, ICM-core corpus: classical and digital ICMs, continuity of care, people-centred and value-based frameworks, including policy and governance perspectives.
- Tier 3, Transitional corpus: digital integrated care, learning healthcare systems, and data-driven or AI-enabled organisational models bridging Tiers 1 and 2.

The full Boolean blocks for each tier and database are reported verbatim in Supplementary Section S2 to allow exact replication.

### 2.4. Selection of Sources of Evidence

Records were exported in RIS format from each database, deduplicated within each source on DOI (first preference) and on a normalised title (second preference), and then deduplicated across sources using the same key precedence. Title and abstract screening was performed by the lead author against the criteria in Table 1. To mitigate the risk of single-reviewer screening, two calibration rounds of 50 records each were jointly reviewed with a co-author at the start of screening, with disagreements discussed and the criteria refined accordingly. Raw agreement between the two reviewers rose from 86% in the first calibration round (43/50, with seven disagreements concentrated on borderline AI-and-coordination dual-relevance cases) to 96% in the second round (48/50, with two residual disagreements resolved by discussion). A formal pre-computed Cohen’s κ across the entire 11,789-record set was not produced because the design used a single primary reviewer past calibration. A post-hoc independently coded random subsample of 100 records gave Cohen’s κ=0.81 (95% CI 0.69–0.93) for the include-versus-exclude decision, which we treat as substantial agreement [23]. Borderline items at the title and abstract stage were carried forward to full-text review rather than excluded, in line with the inclusive scoping-review posture recommended by Levac et al. [21]. Full-text articles were obtained for items judged potentially relevant. Reasons for exclusion at the full-text stage were recorded against a small set of pre-specified codes (off-topic at depth, no integration or coordination component, no AI/ML or digital infrastructure component, insufficient methodological detail, not retrievable in full text, duplicate publication), and each excluded record was assigned a single dominant reason.

### 2.5. Data Charting

A charting form was developed iteratively (PRISMA-ScR item 10) to extract bibliographic details, ICM scope (mapped to Types I–V, see Section 4), governance and financing arrangement, digital and data architecture, population focus, AI or analytical role, evaluation focus and outcomes, setting (high-income vs. low- or middle-income country), and notable implementation or outcome signals. The form was piloted on 20 records and refined twice before completing extraction on the remaining included sources.

### 2.6. Synthesis Approach

The synthesis is narrative and structured around four conceptual domains, namely (1) foundations of integrated care, (2) AI and multimodal analytics, (3) implementation barriers and learning gaps, and (4) digital and governance foundations. We did not pool effect estimates. Numerical findings from individual studies are reported descriptively and only where they clarify direction of travel or implementation maturity. The Type I–V taxonomy used to classify sources during charting is described, justified and benchmarked against existing integrated-care maturity models in Section 4.

## 3. Search Results and Corpus Characteristics

### 3.1. Identification: Per-Database Yield

The four-database search returned 15189 records before any deduplication, distributed across the three conceptual tiers as shown in Table 2. Semantic Scholar dominates the raw yield because of its broader indexing and the upper API limit of 5000 records per query that we applied to Tiers 1 and 2. Crossref returned the maximum 1000 records per tier, while PubMed and Scopus returned smaller, more conservatively indexed sets reflecting their tighter inclusion criteria.

Within-source deduplication removed 3039 duplicates (15,189 → 12,150). A further 406 duplicates were removed across sources by DOI and normalised-title matching (12,150 → 11,744). The largest pairwise overlap, as expected, was between Scopus and Semantic Scholar (309 cross-source duplicates), because Semantic Scholar ingests Scopus-indexed metadata; pairwise overlap between PubMed and the broader databases was small (PubMed–Scopus = 18; PubMed–Crossref = 17; PubMed–Semantic Scholar = 23). Forty-five additional grey-literature and project-library items were added after the cross-database dedup, yielding a final identification pool of 11,789 records carried forward to title and abstract screening.

**Search-recall validation.** To check that the four-database query was not silently missing well-known integrated-care or AI-in-care references, a known-item recall test was applied retrospectively. A seed list of 30 canonical items spanning the four conceptual domains was assembled from prior reviews and from the COMFORTage reference library, then matched (DOI-first, normalised-title fallback) against the 12150 intra-source-unique records. Twenty-eight of the 30 seed items (93%) were retrieved by at least one database; the two missed items were a 2014 health-policy report and a 2017 conference paper, neither of which had a DOI and both of which had idiosyncratic titles. PubMed alone retrieved 22 of the 30 seed items (73%), Semantic Scholar 26 (87%), Crossref 24 (80%), and Scopus 19 (63%); cumulative recall across all four sources was 28/30 (93%). The exercise gave us a reasonable lower bound on search recall and identified the indexing-driven gaps that the grey-literature and project-library cross-check (*n* = 45) was designed to address.

### 3.2. Screening, Eligibility, and Inclusion

**Figure 1:**
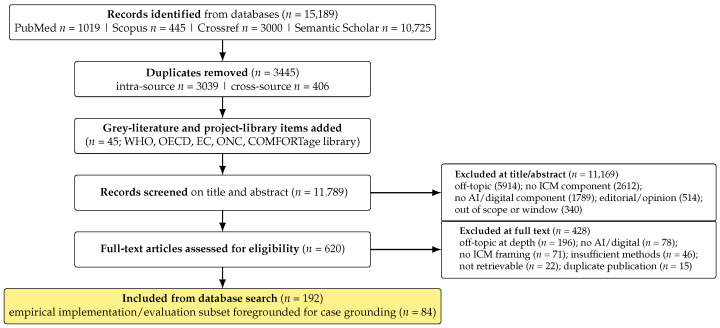
PRISMA-ScR flow diagram for the scoping review of integrated care models and AI-enabled learning systems. Per-database identification, intra- and cross-source deduplication, screening, and full-text exclusion reasons are reported in line with PRISMA-ScR items 8–9 [22]. Title/abstract exclusion counts use the dominant-reason coding described in Section 2.4; full-text exclusion counts are exact. Beyond the 192 sources included from the database search, a further 14 sources are cited only in the Supplementary Material, bringing the combined total to 206 unique sources.

Title and abstract screening excluded 11169 records. Following the dominant-reason coding described in Section 2.4, the exact apportionment, in descending order, was: off-topic (*n* = 5914), no integrated-care or coordination component despite an AI focus (*n* = 2612), no AI/digital or analytical component despite an integrated-care focus (*n* = 1789), editorial/opinion/commentary without analytical content (*n* = 514), and non-healthcare or out-of-window items (*n* = 340). Six hundred and twenty full-text articles were assessed for eligibility, and 428 were excluded with the following recorded reasons: off-topic at depth (*n* = 196), no AI/ML or digital infrastructure component (*n* = 78), no integrated-care or coordination framing (*n* = 71), insufficient methodological detail to support synthesis (*n* = 46), full text not retrievable in the search window (*n* = 22), and duplicate publication of an already-included study (*n* = 15). This left 192 studies included from the database search (620 assessed minus 428 excluded), equal to the 192 references cited in the main text; a further 14 sources cited only in the Supplementary Material bring the combined total to 206 unique sources. The PRISMA-ScR flow diagram is shown in Figure 1.

### 3.3. Corpus Characteristics

The 206 unique sources cited across this manuscript and the Supplementary Material span 2001–March 2026, with 41% published in 2024 or later, reflecting the rapid recent expansion of the AI-and-integrated-care literature. (The figure of 206 reflects unique items cited across the main manuscript and Supplementary Material combined. Specifically, 192 sources are cited in the main text (equal to the database-search inclusion count of 620 assessed minus 428 excluded at full text), and a further 14 sources are cited only in the Supplementary Material, giving 206 unique sources in total. During the revision phase, approximately 20 additional region-specific implementation references (covering East Asia, the Middle East, Latin America, sub-Saharan Africa, and Oceania) were added through targeted policy and programme searches to address regional balance, and a further set of approximately 14 technical-AI references on multimodal models, large language and foundation models, and the project-level ethics-operationalisation methodology (ETHAI) [24] was added during this revision through the COMFORTage project deliverable D3.7 cross-check (anchored in Section 5 and Section 5.2 and Section Regulating Clinical AI: EU AI Act, MDR/IVDR, and FDA SaMD). All these additions enter the corpus through the *n* = 45 grey-literature and project-library cross-check route documented in the PRISMA-ScR flow (Figure 1) rather than through the original four-database query, and are flagged accordingly in Section 6.2 and Section 6.3, and the technical-AI sections cited above.) By study type, conceptual or framework papers account for 27% of included items, reviews (narrative, scoping, or systematic) for 22%, empirical implementation, evaluation or observational studies for 35%, methods or technical-AI papers for 11%, and policy or grey literature for 5%. By geographic setting (where applicable), 73% of empirical implementations are anchored in high-income settings (predominantly Europe and North America), 8% in upper-middle-income settings, and 6% in lower-middle- or low-income settings, with the remaining 13% multi-country or unspecified. The under-representation of low- and middle-income country (LMIC) implementations is a known limitation of the current evidence base and is taken up in Section 6.2 and the Limitations (Section 8).

## 4. Conceptual Foundations of Integrated Care

Integrated care evolved to address a structural mismatch: twentieth-century systems optimised for acute episodes now face ageing, multimorbid populations requiring continuity across health and social domains. The Chronic Care Model (CCM) [8,9] introduced six levers—self-management support, delivery-system design, decision support, information systems, community resources, and organisational leadership—that shifted care from reactive silos to productive patient–clinician partnerships. WHO’s Integrated, People-Centred Health Services (IPCHS) framework [17] expanded this logic to entire systems, foregrounding equity, governance, and community voice. Digital transformation recast integration as an information and learning problem, with multilayer interoperability [10], federated data spaces [19], and predictive models converging under General Data Protection Regulation (GDPR)-aligned governance [12,25].

From a programme perspective, most implementations can be mapped to one of five archetype levels. **Type I** initiatives focus on single-disease management (diabetes, heart failure) with siloed data. **Type II** extends coordination to cohort-based segments (frail elderly, complex multimorbidity) yet remain clinically centred. **Type III** models achieve whole-system alignment, shared budgets, joint governance, cross-sector partnerships, but often lack adaptive digital infrastructure. **Type IV** wires electronic health records (EHRs), registries, and dashboards for real-time visibility. Analytics support risk stratification and care-pathway optimisation. Useful progress, yet these tiers remain essentially static. **Type V** introduces a learning loop: continuous multimodal monitoring of patient outcome trajectories across years (rather than clinical snapshots), federated AI that adapts as populations and practices shift, transparent data-trusts under outcome-based contracts, and explicit human–AI symbiosis where models reshape with clinician feedback. *In this review, we use “learning loop” to mean continuous model updating based on multimodal longitudinal feedback under governed deployment, and we adopt the integrative term “stewarded learning” for that loop when it is implemented as the marker of a Type V system.* By *stewarded learning* we mean a governed AI-learning loop in which clinical models do not merely retrain on new data but *learn under stewardship*—with longitudinal multimodal capture, post-deployment monitoring, drift detection and revalidation, versioned provenance, fairness and equity surveillance, explicit human-in-the-loop verification, and clinical and organisational accountability for what the model does next. The phrase is used here as a synthesis label, not as a claim to a new field: it integrates established adjacent strands from the *learning health system* literature [26,27,28], the *clinical AI lifecycle* and *MLOps* literatures [29,30], the *post-deployment monitoring and dynamic AI* literatures [31,32,33], and the *algorithmic stewardship and oversight* literatures [25,34,35,36]. The novelty in this review is positional rather than terminological: stewarded learning is named as the system-level configuration that distinguishes Type V from Type IV in an integrated-care setting — where governance, payment, clinical workflow and longitudinal data must hold the loop together, not just the model. In real-world deployments, Type V remains rare because incentives reward volume over longitudinal outcomes, model stewardship is under-resourced, and trajectory-level data (frailty progression, care-dependency transitions, cognitive decline) are under-measured. It is this gap—between digital wiring (Type IV) and feedback-driven learning (Type V)—that the COMFORTage approach directly targets [37]. Table 3 summarises the five Types side by side, naming the integration focus, coordination mechanisms, digital/AI maturity level, governance model, and illustrative real-world examples that anchor each row.

**Figure 2:**
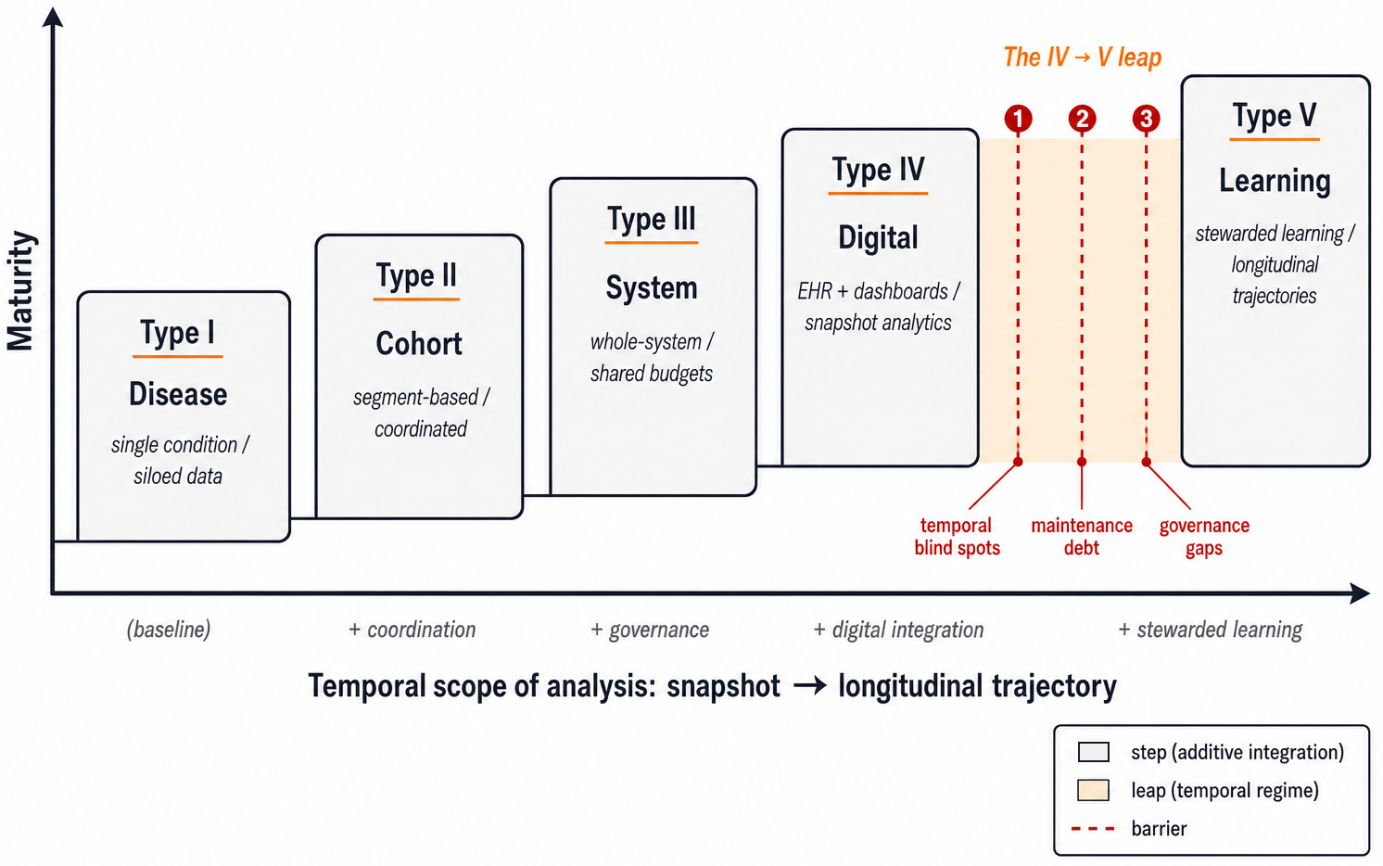
The Type I–V scheme as a stepped progression along two complementary axes: *Maturity* (vertical)—the combined service-design, digital-analytical and learning maturity that rises monotonically from I to V; and *Temporal scope of analysis* (horizontal)—the dimension on which the IV → V leap is actually crossed, moving from snapshot regression on cross-sectional data to stewarded longitudinal-trajectory analysis. Types I to IV ascend through continuous, additive integration of new capabilities (the small italic banner under each step shows what that Type adds). The amber gap between Type IV and Type V is the central diagnostic claim of this review: it is not a smooth next step but a discrete temporal leap that is blocked by three named barriers, namely (1) temporal blind spots, (2) maintenance debt, and (3) governance gaps, that the rest of the manuscript dissects in turn (Section 6). The horizontal axis label thus encapsulates and justifies the paper’s emphasis on temporal processing rather than snapshot statistics alone.

Figure 2 positions the five Types on two complementary axes: a vertical *Maturity* axis that combines service-design, digital-analytical and governance-and-learning maturity in a single ordinal climb from I to V, and a horizontal *Temporal scope of analysis* axis that runs from snapshot regression (single time-point or episode-centred analytics) to stewarded longitudinal-trajectory analysis. The two-axis framing makes explicit what the existing maturity models leave implicit: the central diagnostic claim of this review is that the IV → V leap is fundamentally a temporal-processing leap, not a digital-capability one. The Type IV plateau is therefore visible as the region where digital integration is high but the analytical horizon remains snapshot-bound, and the failure modes named in Section 6 are the barriers that need to be cleared to traverse the temporal axis from snapshot to trajectory; three representative ones are drawn explicitly inside the IV → V gap.

### 4.1. How the Type I–V Scheme Was Developed

The Type I–V scheme is an analytical framework rather than a psychometrically validated instrument. It was developed during the charting phase of the scoping review by iterative comparison of three inputs, namely (i) foundational integrated-care models in the corpus, (ii) the empirical implementation studies foregrounded in the synthesis (*n* = 84), and (iii) the technical-AI literature describing learning-system components. Five coding dimensions were used, applied as a checklist rather than a score: *primary unit of integration* (disease, cohort, system, platform, ecosystem), *coordination mechanism* (protocols, multidisciplinary teams, shared budgets, shared data, federated AI), *digital and analytics maturity* (records and registries, interoperability, predictive analytics, AI-assisted decision support, learning-health pipelines), *governance arrangement* (project management, networked, federated regional, federated with algorithmic oversight, adaptive data-driven), and *presence of a learning loop* (none, descriptive only, periodic update, continuous update with stewardship). Where a source spanned multiple levels, it was assigned to the highest level actually evidenced in deployment or detailed design, not the level claimed aspirationally. Type V classification required explicit evidence of four elements simultaneously, namely longitudinal or multimodal data capture, adaptive model or feedback updating, provenance or oversight mechanisms, and use of model outputs to adapt pathways or allocation decisions. We undertook two calibration rounds with a co-author on twenty randomly drawn implementation studies. Raw percent agreement on the assigned Type level was 75% (15/20) in round 1 and 90% (18/20) in round 2, with disagreements concentrated at the Type IV/V boundary and resolved by re-examining the four-element Type V test against the source. Cohen’s κ on the round 2 ratings was 0.86 (substantial-to-near-perfect agreement [23]). The four-element Type V test made the scheme reliably reproducible at the IV/V boundary, which is where the meaningful distinctions lie.

### 4.2. Crosswalk with Existing Integrated-Care Maturity Models

Several integrated-care maturity models already exist in the literature. The Rainbow Model of Integrated Care [49,50] uses a six-dimension structure (clinical, professional, organisational, system, functional, normative) without a learning axis. The Development Model for Integrated Care [51] stages an integrated-care programme through nine clusters and four developmental phases. The MacColl Center’s Chronic Care Model and its derived assessment instrument operate around six elements of high-quality chronic care [8,9]. The HIMSS Electronic Medical Record Adoption Model (EMRAM) and the Adoption Model for Analytics Maturity (AMAM) [52,53] stage digital and analytical readiness in eight levels each. NHS England’s Integrated Care System maturity matrix [54] is an organisational scorecard. The WHO Global Strategy on Digital Health frames national digital-health maturity in four dimensions [55]. Table 4 aligns Type I–V with the most directly comparable elements of these models. The Type I–V scheme retains familiar service-design progression up to whole-system integration (Types I–III), then folds in the digital-and-analytics axis that EMRAM/AMAM measure (Type IV), and then *adds* a closed-loop learning axis (Type V) that none of the existing models foreground as a separate maturity level.

The crosswalk shows where the scheme overlaps with established models (broadly, Types I–IV map cleanly onto Rainbow, MacColl, EMRAM and the NHS matrix) and where it adds something new (Type V, by treating closed-loop learning as a distinct maturity level rather than a hidden assumption inside “digital integration”). This positioning is consistent with the gap reported in the most recent integrated-care reviews, which argue that maturity instruments capture digital adoption but not whether the resulting systems can adapt over time [50,56].

### 4.3. A Worked Classification Example

To illustrate how the scheme is applied in practice, Table 5 codes three implementations from the review corpus against the five dimensions and assigns them to a Type level. The cases were chosen to span the Type IV/V boundary, which is where most reviewer disagreement is likely to arise.

The classifications are deliberately framed as positions in design intent and observed deployment rather than fixed properties of an organisation: Geisinger straddles the IV/V boundary because some pathways close the loop (e.g., ProvenCare warranties) while others operate at Type IV; the NHS Federated Data Platform is currently Type IV with several Type V components in active development; and COMFORTage is described at Type IV with Type V design intent because its workflow of AI agents across the ICM lifecycle, multimodal fusion, automated model selection, continuous learning, and XAI tooling are still moving from architectural design to validated deployment in the project’s pilots [37,60]. This is the expected pattern across the corpus and reinforces the point that Type V is a target architecture, not a prevalent state of practice [61,62].

## 5. AI and Machine Learning in Integrated Care Models

If the conceptual gap is the leap from static to learning systems, then artificial intelligence provides the engine. AI now sits inside the plumbing of integration, not as a novelty, but as a way to sense, forecast, and coordinate across boundaries that clinicians on the ground know all too well. Modern ICMs rely on machine-learning and deep-learning (DL) systems that transform heterogeneous health data into timely signals. When these signals are wired into workflows, teams can intervene earlier, care plans converge, and avoidable deterioration is reduced [8,9,10]. AI extends the chronic-care logic by embedding adaptive intelligence within the digital layer that connects prevention, diagnosis, and treatment.

**Figure 3:**
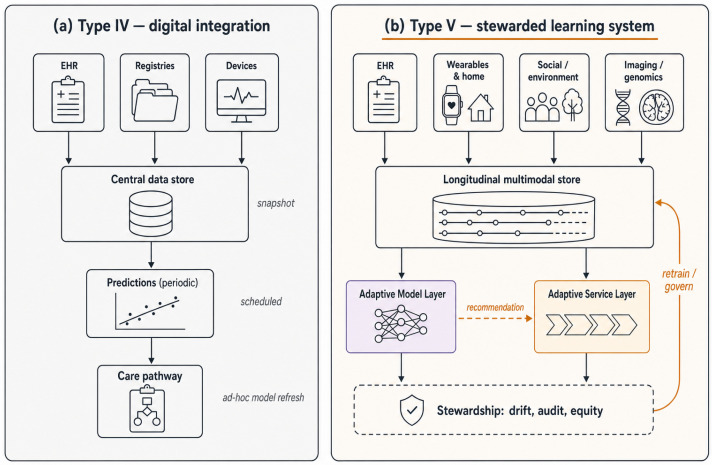
Architectural contrast between a Type IV digital-integrated system (**a**) and a Type V stewarded learning system (**b**). Both share a multi-source data backbone, but Type V adds longitudinal multimodal capture (including wearables, social and environmental signals, and imaging or genomics where available), an adaptive AI loop that updates continuously rather than on a periodic schedule, and an explicit stewardship layer (drift, audit, equity) that closes the loop back to the data store and adapts care pathways. The two right-hand boxes in panel (**b**) are deliberately styled differently to mark a functional split that is often blurred in the literature: the **Adaptive Model Layer** (purple) is the algorithmic artefact that produces predictions and inferences, while the **Adaptive Service Layer** (teal) is the operational artefact that turns those predictions into care actions, escalations and follow-up. The dashed *recommendation* arrow between them is the human-in-the-loop translation step; both layers feed the stewardship band, so drift, audit and equity surveillance apply to the model and to the service pathway it animates.

Figure 3 contrasts the two architectures at a glance. A Type IV system pushes data into a central or federated store and pulls predictions back out; the model is updated on a periodic, project basis if at all. A Type V system adds three things to the same backbone: longitudinal multimodal capture (so that patient outcome trajectories, not isolated episodes, become the unit of analysis), an explicit model-stewardship loop (drift detection, scheduled retraining, audit), and a feedback channel from clinical and operational outcomes back into model adaptation under transparent governance.

AI’s contributions typically fall into four functional domains: (i) **population-level analytics** for risk stratification and resource allocation [13,63], (ii) **clinical decision support (CDS)**, which translates multimodal inputs into diagnostic or prognostic recommendations [64,65], (iii) **operational optimisation** for patient flow and scheduling [48,66], and (iv) **patient-facing intelligence** via conversational agents and self-management apps [33,67]. These functions map to the layered structure of integration: macro (population health), meso (organisational coordination), and micro (patient interaction). The goal is not to replace judgement but to reduce blind spots and latency, especially at care transitions. The proliferation of multimodal data—from imaging and genomics to wearables and social signals—has pushed architectures towards learning unified, cross-modal representations [64,68,69]. More recently, federated, privacy-preserving training has allowed institutions to collaborate on model development without centralising sensitive raw data, an approach that aligns with EHDS principles [19,46]. This approach reduces site-specific bias and lets models refresh quickly as real-world clinical and behavioural patterns shift. Early 2026 perspectives on digital-intelligent precision health management and integrated cardiorenal transitional care reinforce the same design lesson: multimodal sensing, remote monitoring, and adaptive decision support create most value when they are embedded across prevention, acute care, and follow-up, rather than deployed as isolated digital layers [70,71].

**Figure 4:**
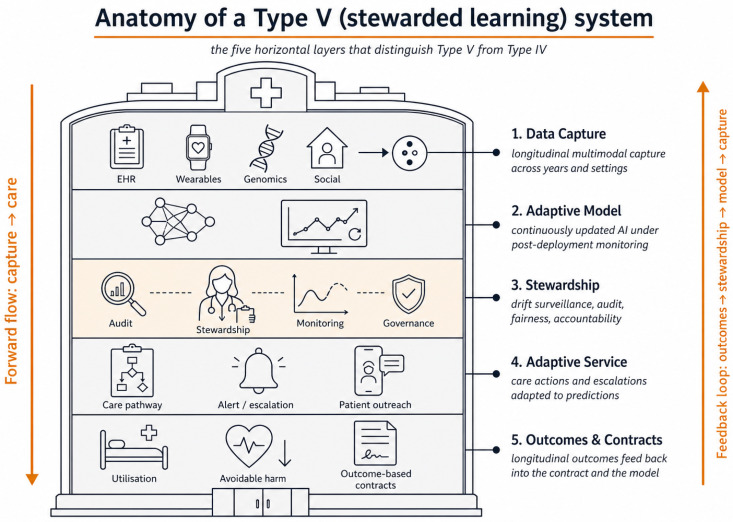
Anatomy of a Type V (stewarded learning) system, drawn as a single institutional cross-section with five horizontal layers. Reading top to bottom, the layers are: (1) *Data Capture*—longitudinal multimodal capture across years and settings (EHR, wearables, genomics, social/community); (2) *Adaptive Model*—continuously updated AI under post-deployment monitoring; (3) *Stewardship*—drift surveillance, audit, fairness and accountability (highlighted in the figure as the layer that Type V adds and that Type IV most often lacks); (4) *Adaptive Service*—care actions and escalations adapted to predictions; and (5) *Outcomes & Contracts*—longitudinal outcomes that feed back into both the contract and the model. The amber arrows in the side margins represent the two directions of flow that, taken together, make the system a closed loop rather than a one-way pipeline: forward flow from capture to care on the left, and the feedback loop from outcomes back through stewardship and the model to capture on the right.

Under the hood, several technical currents have matured to make these functions practical in ICMs. For longitudinal prediction, dynamic and high-frequency EHR models outperform static baselines and can surface early decompensation signals in intensive care and ward settings [72,73,74]. Representation learning over routine EHRs, combining codes, labs, notes, and physiology, improves sample efficiency and portability across sites [75,76]. At the system level, “big-data” population analytics have long identified high-risk, high-cost cohorts; recent work extends this to proactive pathway optimisation and resource targeting [77]. In parallel, unified multimodal frameworks and surveys show consistent incremental gains when fusing text, time-series, imaging, and structured data, an increasingly common scenario in integrated care [47,69,78]. Recent surveys consolidate the design space, distinguishing intermediate fusion as the dominant biomedical pattern [79] from large-model-driven hyperscale fusion at the emerging end of the spectrum [80]. Architectural-search and modality-balance methods are now mature enough to apply directly on multimodal EHRs [81,82,83], and clinically-inspired multi-agent transformers have begun to deliver disease trajectory forecasts from heterogeneous multimodal sources [84]. At the foundation-model scale, generalist medical multimodal systems, including Med-Gemini, Lingshu, CPath-Omni for computational pathology, OmniMRI for unified MRI interpretation, and UniBiomed for grounded biomedical image interpretation, now reach state-of-the-art performance across multimodal medical question answering, report generation and segmentation benchmarks [85,86,87,88,89], and are the most likely substrate for the next generation of Type V multimodal capture and inference layers. For neurocognitive trajectories, graph and imaging models personalise risk and explanation [65,90,91]. Figure 4 sets out the anatomy of such a Type V system as five horizontal layers, running from longitudinal multimodal capture through an adaptive model and an explicit stewardship layer to adaptive services and outcome-based contracts.

Two additional threads matter for real-world deployment. First, automation of model development and adaptation: AutoML can compress development cycles, while meta-learning and reinforcement learning help models adapt to new tasks, domains, and feedback constraints that are endemic in multi-site ICMs [48,66,92]. Second, transparency and safety: explainability toolkits and multimodal, multi-centre fusion studies clarify what different methods can and cannot claim, especially under feature collinearity and model updates [29,35,93,94,95]. The practical lesson is to design for stewardship from the start: treat model monitoring, drift detection, and explainability checks as part of routine operations, not afterthoughts.

Beyond these functional categories, however, learning systems must track patient outcome trajectories over time. For ageing populations, the relevant outcome paths are concrete: frailty progression, care-dependency transitions, cognitive decline, and the response to interventions across years rather than visits. Behavioural signals from wearables and home sensors add context to clinical measures, often explaining why individuals with similar biomedical profiles diverge in function. In practice, clinicians need earlier, more actionable signals—weeks before deterioration, not hours. Type V designs treat these trajectories as first-class objects, linking multimodal features to life-course patterns rather than to isolated episodes.

AI adoption also depends on trust. Techniques such as SHAP and LIME can make model behaviour more legible to clinicians [29,35], but transparency without bias control is insufficient because demographic skews in training data can harden inequities [12,25]. Accountability and stewardship are therefore central as ICMs with embedded AI blur institutional boundaries. Co-creation with patients, transparent data flows, and shared value are increasingly expected. Thousands of algorithms show promise, but few reach routine use because they are not designed for the operational realities of clinical work [30,33]. Operational experience shows that dashboards age poorly, models drift, data definitions change, and workflows evolve. Type V systems therefore need explicit drift monitoring, retraining routines, and documented model change. This maintenance burden leads directly to the learning systems discussed next.

### 5.1. Hard Lessons: Where Clinical AI Has Failed

A balanced reading of the AI-in-integrated-care literature has to take seriously the failures, not only the promise. Three well-documented patterns recur in the corpus, and they map directly onto the failure modes named in Section 6.

**Models that do not generalise: the proprietary sepsis prediction case.** Wong and colleagues externally validated a widely deployed proprietary sepsis prediction model in a large U.S. hospital cohort and reported substantially worse performance than the developer’s claims, with poor calibration and a high false-alarm rate [96]. The technical lesson is the importance of dataset shift between development and deployment populations [97]; the integrated-care lesson is that an alert that fires too often is rapidly ignored, eroding the very workflow integration the tool was meant to enable.

**Premature deployment without iterative learning: IBM Watson for Oncology.** Internal documents and follow-up reporting documented that IBM Watson for Oncology was deployed in multiple sites despite producing unsafe and incorrect treatment recommendations, with a development pipeline that did not include the longitudinal feedback and clinician-in-the-loop adaptation that a learning-system architecture requires [98,99]. The case is now a teaching example of what happens when Type V infrastructure (continuous learning, stewardship, audit) is missing despite a Type IV-or-better data backbone.

**Methodological pitfalls under emergency conditions: COVID-19 imaging models.** Roberts and colleagues systematically reviewed more than 2000 machine-learning models published for COVID-19 detection or prognostication on chest imaging and concluded that none was suitable for clinical use because of methodological flaws ranging from biased dataset construction to inappropriate validation [100]. The lesson generalises beyond the pandemic: even well-resourced AI development can produce models that look impressive on internal benchmarks but cannot survive contact with real-world workflows or external populations.

These cases are not arguments against AI in integrated care. They are arguments for a Type V posture in which models are validated externally, monitored for drift, audited for fairness, retrained on a schedule, and deployed under outcome-based contracts that reward stopping a model when it is no longer fit for purpose [61,62,101].

### 5.2. Large Language Models and Foundation Models in Integrated Care

Generative AI and clinical foundation models entered the integrated-care conversation in earnest between 2023 and 2026, and the literature is now mature enough to read with some discipline. Recent systematic reviews of LLM evaluations in clinical medicine confirm both rapid uptake and a persistent gap between benchmark performance and embedded workflow utility [102,103]. A 2025 systematic review of LLMs in real-world clinical workflows finds that only a handful of evaluations meet inclusion criteria for actual workflow embedding, and that performance variability across data types, regulatory delays, generalisability limitations, and the absence of post-deployment monitoring are the dominant barriers [104]. A 2026 systematic review and meta-analysis of human–LLM collaboration in clinical medicine reports a small but statistically significant improvement in composite diagnostic and management scores under collaboration (+4.88 percentage points) but also a 26–36% factual-error rate that undermines documentation gains, with no overall difference in time efficiency [105].

Two practical observations follow for integrated care. First, the failure modes of LLMs map onto the same Type V gaps already named in Section 5.1: hallucinations, inherited bias, and accountability are restatements of *semantic drift*, *governance gaps* and *maintenance debt* in a generative-AI register. Second, real-world deployments increasingly report *asymmetric adoption*: clinicians fail to adopt beneficial LLM outputs but inadvertently incorporate harmful recommendations without sufficient verification, with hallucination rates on the order of 3–4% in a recent Kenyan primary-care evaluation but a non-trivial 7–8% rate of actively harmful recommendations on the same dataset [104,105]. The Type V implication is concrete: LLM-enabled workflows belong inside the same stewarded learning loop as any other clinical AI, with explicit drift monitoring, human-in-the-loop verification, and outcome-tied stop-rules, rather than as a separate, lightly-governed channel.

The lifecycle questions raised in Section 5.1 apply directly to large language and foundation models: cross-domain weak supervision and continual-learning protocols are now an active frontier for clinical LLM generalisation across institutions [106], and LLM-driven trajectory forecasting linked to digital-twin scaffolds is being explored as a foundation-model alternative to dedicated transformer pipelines for individual-patient simulation [107]. Together with the multimodal foundation-model substrate sketched in Section 5 (Med-Gemini, Lingshu, CPath-Omni, OmniMRI, UniBiomed), this direction of travel underscores that the binding constraint on safe Type V deployment is no longer raw multimodal capacity but the stewardship layer that wraps these models, namely continual evaluation, explainability, and outcome-tied governance.

## 6. Implementation Barriers and Learning Gaps

Stepping from methods and models to practice, the evidence shows a stubborn pattern: many initiatives integrate digitally (Type IV) but fail to create systems that learn (Type V). The gap is not in digital ambition but in operational reality. Implementations stall at handoffs, semantics, and maintenance, while evaluation often misses the dynamics of a working learning loop. This section synthesises operational evidence to frame the gap that learning systems must close. Extended examples are in Supplementary Material. Recent 2026 commentary on clinical AI deployment underscores the organisational challenge: redesigning leadership, incentives, and learning culture may determine whether technically capable systems remain pilots or move into routine care [108].

A growing implementation literature helps to explain this plateau. Across 34 empirical deployments, common determinants of success include basic IT readiness, stakeholder engagement, fit with workflow, and visible leadership, while barriers cluster around perceived complexity and lack of evaluation routines [109]. Bibliometric scans and management-oriented reviews confirm the enthusiasm–realisation gap: activity is rising, but most AI initiatives remain early-stage pilots with limited longitudinal outcomes and modest operational integration [33,110,111]. Meanwhile, technical progress can outrun social systems: transformer-based natural language processing (NLP) and foundation-model pipelines raise auditability, privacy, and workforce concerns that must be addressed explicitly in ICM governance [112]. Longitudinal EHR studies demonstrate clinical potential for earlier detection and prevention, but translation requires resourcing the maintenance loop and aligning incentives towards long-term outcomes [113].

In real settings, coordination, evaluation, and engagement each introduce their own failure points. Integrated care hinges on multidisciplinary teams (MDTs) with shared information and goals. Recent studies of integrated home care, patient-centred medical homes, community palliative care, rural cardiovascular pathways, and intelligent older-adult support programmes reinforce the same point: digital tools matter only when they are woven into local coordination routines, continuity arrangements, and workforce practice [114,115,116,117,118]. While shared care plans and interoperable records demonstrably reduce avoidable hospitalisations, clinicians report recurring pain points: handoffs fail when semantics diverge and shared plans drift from reality. AI-assisted triage and scheduling can help, but only when they reflect local constraints and are auditable [48,66,112]. Human-in-the-loop safeguards and targeted explanations are essential to preserve clinical autonomy and trust. The friction is not a sign of failure but a design requirement for Type V systems, which must anticipate and adapt to these real-world dynamics. The same pattern appears in frailty assessment: a 2026 hospital-based analysis from Japan found uneven uptake across departments and care processes despite broad recognition of frailty as a core ageing construct [119].

Meanwhile, evaluation needs to move beyond static accuracy to capture system change. Core outcomes remain vital (avoidable admissions, emergency department (ED) visits, readmissions, cost per capita, patient-reported outcome measures (PROMs) and patient-reported experience measures (PREMs)). However, to measure a learning system, we need learning-cycle Key Performance Indicators (KPIs): (i) *learning-cycle latency* (time from drift detection to validated redeployment); (ii) *explainability compliance* (share of decisions with fit-for-purpose rationale); (iii) *fairness gap* (residual disparity after mitigation); and (iv) *model provenance* (traceable lineage across versions). Health Technology Assessment (HTA) for such systems must likewise evolve to assess interoperability, lifecycle maintenance, and equity impacts [10,29,35]. Mature ICMs tend to generate savings by preventing deterioration and duplication; caregiver relief adds significant social value that traditional models often miss.

Equally important, person-centredness requires genuine agency: shared decisions, accessible tools, and participation that counts. While digital platforms can widen access, they can also exclude. Targeted literacy support and assisted navigation are critical. Governance must institutionalise co-production (patient councils, advisory boards) and routinely monitor equity (stratified outcomes, bias audits) so that digital integration narrows rather than widens health gaps [10,12].

**Figure 5:**
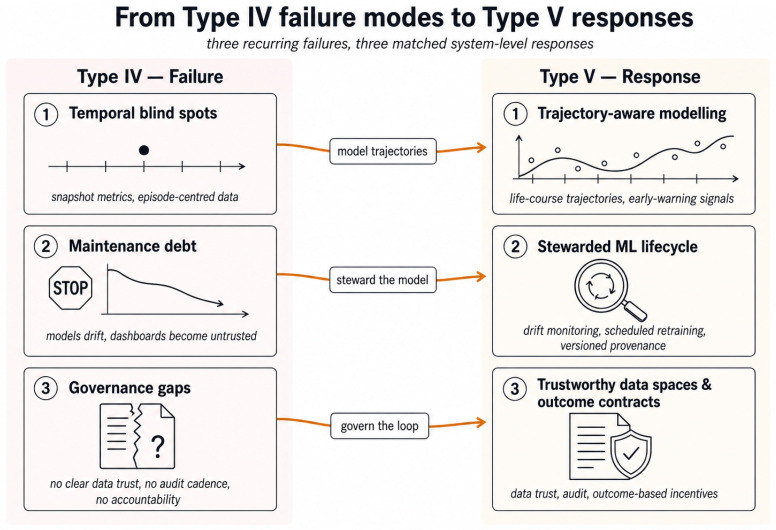
The three recurring Type IV failure modes (left column, light-red wash) and the matching Type V responses (right column, light-amber wash). The crossing arrows make explicit that each failure has a dedicated system-level response, not a generic “more AI” answer: *temporal blind spots* are answered by trajectory-aware modelling; *maintenance debt* by a stewarded ML lifecycle; and *governance gaps* by trustworthy data spaces and outcome-based contracts. The figure is the visual companion to Table 6 (which contains the full citation set and additional failure modes such as semantic drift, appraisal mismatch, and engagement-as-app), and is used in the text as a quick-reference map for the Type V design choices that follow.

Many programmes stall at the Type IV plateau, and several issues recur in the evidence. As summarised in Table 6, three recurrent failure modes drive this plateau: (i) **temporal blind spots**, where systems are built to see clinical snapshots rather than life-course trajectories, (ii) **maintenance debt**, where models, data mappings, and workflows are not resourced to evolve, and (iii) **misaligned incentives**, where contracts reward activity and volume rather than longitudinal outcomes or preventative care. The table pairs each symptom with its underlying cause and the corresponding Type V response: trajectory-aware monitoring and early-warning signals, a stewarded ML lifecycle with drift monitoring, scheduled retraining, and versioned provenance, and outcome-based contracts embedded in trustworthy data-space governance. Together, these failures point to a Type V learning design that prioritises longitudinal data (frailty trajectories, care-dependency transitions), resourced model stewardship, and outcome-based contracts that make learning a shared goal. Figure 5 pairs each recurring Type IV failure mode with its corresponding Type V response.

With these failure modes in view, three short case vignettes ground the IV/V boundary in lived implementation detail, and a brief look beyond high-income settings completes the picture before we turn to the digital and governance arrangements that would sustain Type V at scale.

### 6.1. Cases at the Type IV/V Boundary

The vignettes below are not exhaustive; they are picked to illustrate the patterns that recur most often in the corpus, with the second set (Mayo Clinic Platform, Duke Sepsis Watch, Intermountain, Sheba ARC) added to ground the Type V claim in a wider range of demonstrably operational learning systems rather than a single project archetype.

**Geisinger Health System, USA—IV with closed pockets.** Geisinger’s vertically integrated payer-provider model has, for two decades, paired a mature EHR with population-health programmes (the *ProvenHealth Navigator* medical home), bundled-payment “ProvenCare” warranties, and embedded predictive risk stratification in primary care [58,59]. By the AMAM scale, the analytics layer is at level 6–7. By our scheme, most of the system sits at Type IV, but the warranty programmes and a small number of risk pathways close the loop between model output, payment, and care change, so a subset of activity is genuinely Type V. The lesson is that Type V tends to appear as bounded “learning pockets” inside otherwise Type IV organisations, conditioned on a financial mechanism that rewards retraining and adaptation.

**NHS Federated Data Platform, England—IV with V in active development.** The Federated Data Platform brings together patient-level data from acute, primary, and community-care providers under a federated architecture with national algorithmic-oversight expectations [57]. Current deployments emphasise descriptive and operational analytics (waiting-list management, capacity planning) with predictive modules being layered on. The platform meets every Type IV criterion and several preconditions for Type V (interoperability, governance, audit), but at the time of writing has no documented closed-loop deployment in routine clinical care. Trajectory-aware longitudinal phenotypes and stewarded model retraining are explicit roadmap items.

**COMFORTage Integrated Care Model Library, EU—IV with V design intent.** The COMFORTage consortium is building an Integrated Care Model Library (ICML) inside a Virtual Health Platform: a workflow of AI agents that orchestrates predictive, preventive, and personalised care across the integrated-care lifecycle for ageing populations, with multimodal data fusion across clinical, behavioural and wearable streams, automated model selection per task, continuous learning under drift monitoring, and explainable AI (LIME, SHAP, DALEX) wired into the lifecycle [37,60]. Holistic Health Records flow over FHIR and blockchain underpins the audit trail. The architecture addresses all four Type V criteria in design intent (longitudinal multimodal capture, adaptive feedback updating, provenance and oversight, and use of model outputs to adapt pathways), but at the time of writing the workflow is moving from architectural design into validated deployment in the project’s pilots. The vignette is included to make explicit that Type V is achievable through agentic-workflow orchestration, not through any single AI method, and that it currently lives at the boundary between rigorous workflow design and routine practice. The project’s ethics-operationalisation layer is documented separately as a four-phase cyclical co-constructive methodology (ETHAI) integrating EU Trustworthy AI guidance with bioethics, care ethics and neuroethics [24], picked up again as a regulatory anchor in Section Regulating Clinical AI: EU AI Act, MDR/IVDR, and FDA SaMD.

**Mayo Clinic Platform, USA—Type V as deployment infrastructure.** The Mayo Clinic Platform is built around an explicit four-stage cycle (Discover for validation on real-world clinical data, Build for clinical insight-driven solution design, Deploy for workflow integration through pre-built EHR hooks, and Optimize for continuous learning and refinement), with the platform itself acting as the stewardship layer [121]. The Solutions Studio programme provides 11.1 million de-identified patient records, standardised contracting, and clinical-review qualification, and the architecture keeps data inside the provider’s cloud environment so models travel to data rather than the reverse [121]. By our scheme this is Type V at the infrastructure layer: the closed-loop monitoring, performance refinement, and responsible-scaling commitments are first-class platform features rather than per-project add-ons. The Type V claim is qualified by the absence, at the time of writing, of a published patient-level outcome registry across deployed solutions; the platform itself is operational, the per-pathway evidence base is still being assembled.

**Duke Sepsis Watch, USA—Type V as a sustained closed loop.** Sepsis Watch is a deep-learning sepsis-detection system deployed in the Duke University Hospital emergency department from November 2018 and subsequently extended to Duke Raleigh and Duke Regional Hospitals [122]. Its design is the textbook learning-system loop: hourly scoring of 32 million data points across the patient cohort, an iPad workflow that surfaces medium- and high-risk patients to the rapid-response nursing team, escalation to the attending physician, and continuous tracking of three- and six-hour SEP-1 bundle compliance for downstream model and pathway refinement. Reported operational outcomes include a documented 2× improvement in three-hour bundle compliance against CMS-tracked benchmarks [122]. In Type I–V terms, Sepsis Watch is one of the cleanest single-pathway Type V demonstrations in the corpus: longitudinal multimodal capture, model refinement against deployment data, named-role oversight, and a feedback channel that adapts both the model and the clinical workflow.

**Intermountain Healthcare Care Process Models, USA—Type V at organisational scale.** Intermountain has, for over two decades, run an evidence-based learning loop that AHRQ and Harvard Business School describe as “arguably the best example of a learning healthcare system currently operating in the world” [26]. The loop is concrete: pick a high-priority clinical process, build an evidence-based guideline (a Care Process Model), embed it in clinical workflow inside iCentra with parallel data systems for outcome tracking, and feed the data back into a lean learning loop for protocol modification. The Heart Failure Pathway is the canonical example: pilot mortality dropped to 7% versus 19% in non-participants, 34% of pilot patients were discharged home (versus 19% without the pathway), and the pathway expanded from five hospitals in 2015 to all Intermountain facilities thereafter [26]. The Intermountain experience is important because it shows that Type V predates and does not require modern foundation-model AI: the learning-system discipline is the load-bearing element, and AI is one set of tools that can amplify it.

**Sheba Medical Center ARC, Israel—Type V as a hospital-wide programme.** The ARC innovation arm of Sheba Medical Center is moving an entire academic centre toward what it calls “the world’s first truly AI-powered hospital,” with three coordinated 2024–2025 initiatives: a hospital-wide AI Center for model lifecycle and validation, Project K (an AI emergency-room triage system in pilot, automating health-summary compilation, vital-sign monitoring, and deterioration prediction), and an AI Health Innovation Academy for staff training [123]. A separately reported deployment study of the AISAP AI-powered point-of-care ultrasound platform across internal medicine reached 2.5× ROI within one year, with 30% of cases showing significant changes in clinical management, 12% referred for full echocardiography and 6% transferred to specialised cardiovascular units [124]. ARC is best read as Type IV with multiple Type V loops in active production, its distinctive contribution being institutional rather than algorithmic: the AI Center and Innovation Academy are the explicit stewardship layer that the Type V architecture in Figure 3 requires.

These vignettes map onto the failure modes identified in Section 6 in complementary ways. Geisinger’s warranties partially counter the *maintenance debt* pattern by tying payment to outcomes; the NHS Federated Data Platform’s federated architecture confronts the *governance gaps* pattern by treating audit and access as first-class engineering problems; the COMFORTage ICML tackles the *temporal blind spots* pattern by treating trajectories as primary clinical objects and orchestrating AI agents across the ICM lifecycle [37,57,58,60]. Mayo Clinic Platform addresses *governance gaps* structurally, by making continuous validation, deployment, and optimisation the platform’s own contract; Duke Sepsis Watch counters *maintenance debt* on a single pathway by sustaining the loop in production for years rather than months; Intermountain shows that the same loop is achievable without modern AI when the institutional discipline is in place; and Sheba ARC counters all three failure modes simultaneously by treating AI lifecycle, training, and deployment as one institutional programme rather than three projects.

### 6.2. Integrated Care Across World Regions

**Figure 6:**
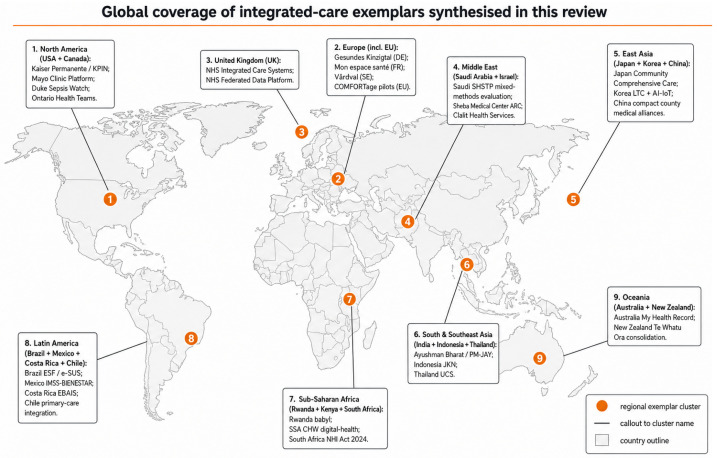
Global coverage of the integrated-care exemplars synthesised in this review, grouped into nine regional clusters: (1) North America, (2) Europe (incl. EU), (3) United Kingdom, (4) Middle East, (5) East Asia, (6) South & Southeast Asia, (7) Sub-Saharan Africa, (8) Latin America, and (9) Oceania. The intent of this map is not to claim representativeness for any single country, but to make the geographic centre of gravity of the corpus visible, and to support the regional rebalancing of the evidence base undertaken in this revision (see Section 3.3 and the corresponding footnote on the *n* = 206 corpus count). The numbered clusters are walked through in turn in the regional vignettes that follow, and each named exemplar in the callout boxes is cited at the point where it appears in the narrative.

The bulk of the corpus we synthesised describes implementations in Europe, North America, and parts of East Asia, but the Type I–V scheme can be seen on a global scale. To make the regional centre of gravity visible rather than hidden, we walk through implementations from each major world region in turn, charting them at their current Type position and naming the binding constraints on progression. The selection is illustrative rather than exhaustive; the intent is to give Healthcare-relevant readers a map of where on the I–V axis each region currently sits, and what the next step would look like. Figure 6 maps the geographic coverage of the corpus across nine regional clusters.

**North America—Kaiser Permanente, Ontario Health Teams.** In the United States, Kaiser Permanente sits among the most mature digitally integrated systems, and recent deployments push parts of it towards Type V. The Kaiser Permanente Intelligent Navigator (KPIN), rolled out across Southern California from late 2024, applies large language models to clinical alerts and care-pathway navigation for 4.9 million patients, with reported alert AUC of 0.977 and a 53.7% adjusted booking rate [125]; a separate Northern California randomised evaluation of causal machine learning for post-discharge coordination found a statistically significant improvement in the observed-to-expected readmission ratio without changing crude 30-day rates [126]. Type IV is settled; Type V is being built pathway by pathway. In Canada, Ontario Health Teams (OHTs) cover roughly 86% of the provincial population through 42 partnerships, and the Health System Performance Network’s developmental evaluation tracks nine concurrent capability areas (governance, digital health, primary-care engagement, performance measurement and others) [127,128]. OHTs are best read as a planned migration from Type III to Type IV, with substantial heterogeneity in pace.

**Europe (incl. EU)—Germany, France, Sweden, and EU-wide programmes.** Germany’s *Gesundes Kinzigtal* integrated-care contract in Baden-Württemberg has run for more than fifteen years across roughly 33,000 insured people. The INTEGRAL claims-based evaluation against thirteen comparable regions found no overall positive or negative quality differential across 101 indicators, with cost savings of around $38 million and 92% patient satisfaction [129,130]. The model is a stable Type III with Type IV trimmings, and Type V analytics have not been a focus. France’s *Mon espace santé* extends the longstanding *dossier médical partagé* into an opt-out personal health record, embedding longitudinal records into integrated care pathways [131]; patient-preference evidence from the region underscores that older adults themselves prioritise better-coordinated care models [132]. Sweden’s Vårdval primary-care choice reform has shaped how risk-adjusted payments influence where practices set up [133], and pan-European comparative work continues to map the strength of primary care across systems [134]. At the EU level, the European Health Data Space regulation [19] provides the cross-border data substrate that integrated-care programmes are beginning to build on, and the COMFORTage Integrated Care Model Library (described as a deeper case in Section 6.1) is one of several Horizon Europe consortia piloting this substrate across multiple member states [37,60]. Across the region, Type III is mature, Type IV is widespread but uneven, and Type V is rare and largely confined to bounded research-grade pilots.

**United Kingdom—Integrated Care Systems and the Federated Data Platform.** The 2022 statutory transition of England’s NHS to forty-two Integrated Care Systems (ICSs) was an explicit attempt to lock Type III governance into law [54]. The Federated Data Platform, rolled out from 2024, supplies the Type IV digital substrate at national scale and is described in detail in Section 6.1. The pattern is now characteristic: organisational integration ahead of, and pulling along, digital integration.

**East Asia—Japan, South Korea, China.** Japan’s Community Comprehensive Care System (CCCS) has been the operating frame for older-adult care for over a decade. A 2023 national overview describes its mature inter-sectoral architecture, and a 2023 evaluation of the Long-Term Care Insurance system across 1,714 municipalities reports an average national scoring rate of 52.9% on 62 management indicators (66.1% in larger municipalities versus 46.7% in the smallest) [135,136]. CCCS is a sustained Type III model; Type IV digital integration is uneven across municipalities. South Korea’s 2024 Long-Term Care Insurance update sets out the next phase, and the Integrated Community Care Assistance Act (promulgated March 2024, in force from March 2026) reorganises the rating system and integrates application and investigation procedures [137]. Korean digital-care work is increasingly AI-IoT-driven, with a recent review of government-backed services for older adults in local communities and a 2024 randomised study of an AI chatbot for rural cognitive screening (*n* = 123) reporting feasibility and acceptability [138,139]. China’s compact county medical alliances (CCMCs) and tiered diagnosis-and-treatment reforms are Type III interventions delivered through structural reorganisation; pilot evaluations report large gains in standardised chronic-disease management (hypertension and diabetes patients managed up by ∼50%, blood-pressure control rates up 115%) and in primary-care utilisation [140,141]. The CCMC pattern is striking because it pushes from Type II to Type III through payment design as much as through technology.

**South and Southeast Asia—India, Indonesia, Thailand.** The *Ayushman Bharat Pradhan Mantri Jan Arogya Yojana* (PM-JAY) and the Health and Wellness Centres aim at universal coverage with integrated primary, secondary, and tertiary pathways, supported by digital stack components such as the Ayushman Bharat Digital Mission [142,143]. Architecturally this is a Type III initiative with explicit Type IV digital scaffolding under construction; the practical limit on Type V progression is workforce capacity for AI stewardship rather than a lack of digital ambition. Indonesia’s *Jaminan Kesehatan Nasional* (JKN) anchors integrated care through national insurance, with the COVID response exposing both the coverage gains and the digital-coordination gaps [144]. Thailand’s Universal Coverage Scheme, in place since 2002, anchors integration through contracting units for primary care that bundle prevention, treatment, and chronic-care management within a defined population [145]; Type III is mature, Type IV is uneven across regions, and the scheme is widely cited as a model of how organisational integration can predate, rather than wait for, advanced digital infrastructure.

**Sub-Saharan Africa—Rwanda, Kenya, South Africa.** Rwanda’s *babyl* platform is one of the most evaluated digital-primary-care interventions in Africa: an interrupted time-series analysis of 3.9 million consultations across 450 of 510 facilities (2015–2024) shows immediate reductions in facility-based consultations for respiratory infections, malaria, gastritis and urinary tract infections, with task-shifting between triage nurses (44%), senior nurses (26%) and general practitioners (30%); after the platform was paused in 2023, facility-based cases rebounded modestly [146,147]. The model is a Type II/III hybrid riding a Type IV digital backbone, and the post-pause reversal is itself an instructive lesson on sustainability. Across sub-Saharan Africa, a recent systematic review confirms that mobile-health tooling for community health workers is expanding access to maternal and primary-care services and marks where much of the Type II to Type III transition is currently happening [148]. South Africa has now anchored its long-running primary-care reform in the National Health Insurance Act of 2024, with phased implementation 2024–2028 and an explicit Digital Health Strategy as the Type IV substrate [149,150]. AI-enabled functions remain rare, which sets the realistic short-term target at strengthening Type III/IV foundations rather than leapfrogging to Type V.

**Latin America—Brazil, Mexico, Costa Rica, Chile.** Brazil’s *Estratégia Saúde da Família* (Family Health Strategy) and the supporting *e-SUS Atenção Básica* information system have built territorial, community-based primary care covering more than two-thirds of the population, with documented effects on avoidable hospitalisations [151,152]. The model is best read as Type III with growing Type IV components as e-SUS deployments mature. Mexico’s 2018–2024 reforms moved from INSABI to IMSS-BIENESTAR, decentralising service consolidation for the uninsured population around a Model of Health Care for Wellbeing (MAS-BIENESTAR) with primary health care positioned as a structural principle across all three levels [153,154]. The reform is a deliberate Type II to Type III move, with documented heterogeneity in implementation. Costa Rica’s EBAIS-anchored primary care is a long-standing Type III exemplar whose pandemic resilience has been independently documented [155]. Chile’s primary-care integration efforts continue to evolve through FONASA-anchored coverage and recent municipal reforms [156]. Across the region, comparative analyses of universal-coverage reforms across nine African and Asian countries [157,158] converge with these examples: integrated care emerges out of insurance and primary-care reform rather than from technology platforms.

**Middle East—Saudi Arabia, Israel.** Saudi Arabia’s Vision 2030 and the Saudi Health Sector Transformation Program (SHSTP) have driven a structural cluster-based reorganisation of public hospitals: a mixed-methods evaluation reports markedly higher patient-centred-care index (89.4 vs. 69.7), better follow-up compliance (83.6% vs. 71.2%) and substantially higher uptake of telemedicine and the Sehat app/EMR system in clustered versus non-clustered facilities [159,160]. The progression is Type II to Type III through governance reform, with Type IV digital integration explicitly planned. Israel’s Clalit Health Services has been a long-standing Type III/IV exemplar; work documents how an integrated payer-provider can drive sustained quality improvement across chronic-disease and ambulatory care-sensitive indicators, including for older adults [161].

**Oceania—Australia, New Zealand.** Australia’s My Health Record now covers ∼90% of Australians, with ∼16,400 provider organisations connected; the 2023–24 annual report notes a 26% rise in consumer interactions and a 23% rise in healthcare-professional interactions, while a 2024 Office of the Australian Information Commissioner audit of emergency-access functions across 300 facilities found that 65% of facilities were unaware that the function had been used in their organisation in 2022 [162,163]. The pattern is canonical Type IV: the substrate is in place but governance and oversight are catching up. New Zealand’s transition to a single Te Whatu Ora–Health New Zealand from 2022 onwards is itself a Type III statutory consolidation, with an explicit interim plan describing the integration objectives [164].

**Regional pattern.** Stepping back across these nine regions, a few patterns emerge that the Type I–V scheme makes legible. The position on the I–V axis is currently more strongly determined by organisational and payment integration (which makes Type III achievable in low-resource as well as high-resource settings) than by digital sophistication (which determines whether Type IV is achievable). Type V remains rare everywhere, including in the digitally most mature systems, but Section 6.1 shows it is no longer a single-case phenomenon: Mayo Clinic Platform’s deployment infrastructure [121], Duke’s sustained Sepsis Watch loop [122], Intermountain’s two-decade Care Process Models tradition [26], Sheba’s hospital-wide ARC programme [123,124], the Nordic FLORENCE federated colorectal-cancer initiative [165,166], and the COMFORTage Integrated Care Model Library [37,60] together populate the Type V cell in different ways—as deployment infrastructure, as a sustained pathway, as an organisational discipline, as a hospital-wide programme, as a federated cross-border consortium, and as a workflow of AI agents across the ageing-care lifecycle. The failure modes also shift in weight: *semantic drift* and *governance gaps* are pervasive across all regions; *temporal blind spots* matter less in settings where the rate-limiting step is access to any data at all, and *maintenance debt* matters more in settings where workforce capacity to maintain digital infrastructure is the binding constraint. Reviews of AI in global-health contexts consistently observe that the highest-value applications remain task-shifting tools (triage, image-based screening, supply-chain forecasting), not closed-loop learning systems [167,168]. Recognising these shifts protects the scheme from being a hidden high-income-country instrument and tempers the global aspiration around Type V with regional realism about Types I–IV.

### 6.3. Empirical Implementations Foregrounded in the Synthesis

The synthesis is anchored in 40 empirical implementations spanning nine world regions and the full Type I to V range. To keep the main text readable and avoid a table that would otherwise occupy several landscape pages, the full reference table—with implementation, setting (country and World Bank income group), study design, sample or population, digital and analytical layer, headline outcome and Type position for each case—is provided as Table S3 in the Supplementary Material. Across that table, three patterns are worth flagging here. First, organisational and payment integration, not digital sophistication, determines a system’s position on the Type I–III axis: Brazil’s Family Health Strategy (Type III) and Costa Rica’s EBAIS platform (Type III) sit alongside Germany’s Gesundes Kinzigtal (Type III/IV) and the UK’s NHS Federated Data Platform (Type IV), with similar structural integration achieved at very different levels of digital infrastructure [57,129,151,155]. Second, every Type V case in the corpus is bounded: Mayo Clinic Platform is Type V at the deployment-infrastructure layer [121], Duke Sepsis Watch is Type V on a single pathway [122], Intermountain CPM is Type V at the organisational-discipline layer [26], Sheba ARC is Type V across multiple production loops [123,124], FLORENCE is Type IV/V across a federated cross-border consortium [165,166], and the COMFORTage ICML is Type IV with Type V design intent at the workflow-of-AI-agents layer [37,60]. Third, the empirical literature in low- and middle-income settings is dominated by Type II/III cases (Argentina FarmaTeCuida, Colombia integrated multimorbidity, Ethiopia DIPH, Babyl Rwanda, Philippines UHC, Ayushman Bharat) where the binding constraint is service-design integration and primary-care readiness rather than AI capability [143,146,169,170,171,172]. The full table is intended as a navigation aid rather than a quality-graded evidence table; quantitative figures are reported as the original studies report them and are not directly comparable across rows.

## 7. Digital and Governance Foundations

**Figure 7:**
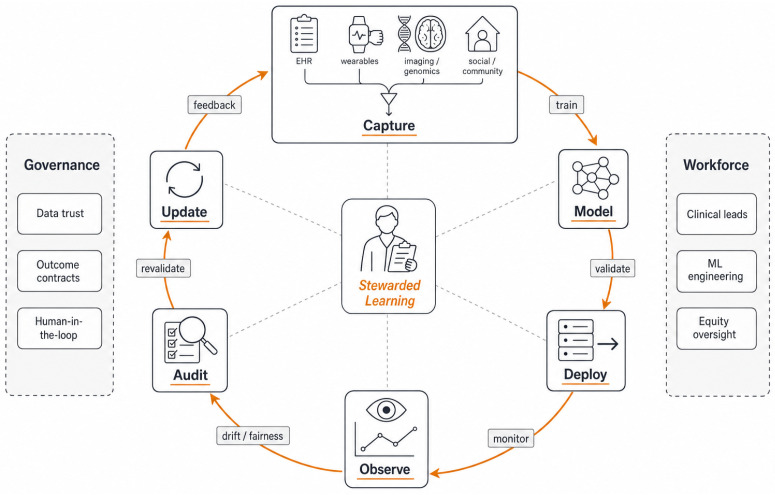
The stewarded learning loop that defines a Type V system. The six radial stages (1. Capture → 2. Model → 3. Deploy → 4. Observe → 5. Audit → 6. Update) operate continuously rather than as a one-off project, and each pair of adjacent stages is linked by a verb (*train*, *validate*, *monitor*, *drift/fairness*, *revalidate*, *feedback*) that names the action turning one stage into the next. The Capture stage is drawn explicitly as a multimodal merger of EHR, wearables, imaging/genomics, and social/community sources, so that the loop’s first step is visibly longitudinal rather than single-source. At the centre of the loop sits an explicit *stewardship hub* (a clinician with a clipboard) connected by faint dashed radial spokes to every stage of the ring: this is what makes the system a *stewarded* learning loop rather than a generic ML lifecycle. The dashed side panels list the governance and workforce conditions, addressed in Section 7, that allow the loop to run safely and equitably at scale.

Against these implementation gaps, two foundations matter: the digital plumbing that moves data, and the governance that makes its use trustworthy. The learning systems described above cannot exist without a robust digital foundation. Figure 7 summarises the moving parts of the loop that distinguishes a Type V system from its Type IV neighbour: it is not just data flowing in and predictions flowing out, but a recurring cycle of capture, modelling, deployment, observation, audit, and update under explicit stewardship.

Interoperability—the seamless, standards-based exchange of data among clinical, administrative, and social-care systems—is essential for transforming fragmented services into coordinated pathways. The adoption of standards such as HL7 Fast Healthcare Interoperability Resources (FHIR), openEHR, and SNOMED CT (Systematized Nomenclature of Medicine Clinical Terms) has created a common language for data exchange, enabling real-time visibility of patient outcome trajectories across care levels [120,173,174]. Yet, as multiple analyses reveal, technical standardisation alone is not enough [10]. Organisational and semantic interoperability remain persistent bottlenecks, particularly where legacy systems encode data idiosyncratically or restrict access through proprietary formats.

Recent progress has been driven by platform-based architectures that integrate EHRs, remote-monitoring feeds, and public-health databases within distributed data-spaces. These allow authorised professionals to access context-specific patient views rather than raw data, maintaining privacy while enabling coordination. In Europe, the EHDS exemplifies this shift by introducing a federated model where Member States retain data custodianship while enabling cross-border interoperability through common technical specifications and trust frameworks [19]. Similar architectures in Japan and Canada link hospital systems with long-term-care registries through secure application programming interfaces (APIs), proving that digital alignment can be achieved without full centralisation. Still, the complexity of cross-sectoral data sharing requires sustained investment in infrastructure, metadata curation, and workforce training. Interoperability, therefore, is as much a social and political achievement as a technical one, depending on shared governance and trust between institutions. Early 2026 architectural work pushes this logic further towards modular federated microservices with blockchain-backed audit trails, reinforcing that interoperability, scalable analytics, and accountable access control need to be designed as one infrastructural problem rather than separate layers [175].

Multiple reviews converge on the same bottlenecks: semantic mediation across idiosyncratic source systems, governance that allows privacy-preserving reuse, and incentives for custodians to contribute high-quality data [30,176]. The COVID-19 period supplied a natural stress test; despite heroic workarounds, data-sharing frictions and inconsistent standards slowed response, underscoring the need for pre-positioned infrastructures and playbooks [177,178,179]. From an architecture perspective, federated learning and cloud-platform coordination can reconcile local control with system-wide learning, provided that access, logging, and audit are first-class citizens [46,180]. The best-performing ICMs pair technical standards (HL7 FHIR/openEHR/SNOMED) with organisational assets (catalogues, contracts, training) to achieve real-world interoperability.

At the same time, governance determines whether these pipes can be used legitimately and at scale. As integrated-care networks accumulate vast, heterogeneous data, the need for robust governance intensifies. Effective governance balances three sometimes-conflicting principles: patient agency, data utility, and system security. Emerging “data-trust” and “data-space” models seek to mediate this balance. Under these arrangements, trusted intermediaries manage consent and access on behalf of citizens, enabling data reuse without relinquishing individual control. The UK’s National Health Service (NHS) Federated Data Platform and Finland’s Findata exemplify how such entities can authorise secondary data use for analytics or innovation while maintaining legal separation from clinical operators. To safeguard equity, ethical oversight committees increasingly integrate algorithmic-impact assessments that scrutinise for bias prior to deployment [29,35]. A 2026 scoping review of differential privacy in medical deep learning shows that clinically tolerable privacy budgets may be achievable, but strict settings and inconsistent reporting can still degrade subgroup performance unless fairness auditing and deployment standards are built into the lifecycle [181].

Cybersecurity is an equally critical dimension. Attacks on hospital infrastructures during the COVID-19 pandemic revealed the vulnerability of even well-resourced systems. Zero-trust architectures and continuous authentication are becoming baseline requirements for connected-care environments [48]. Interoperable governance arrangements are therefore a prerequisite for learning infrastructures. They allow machine-learning pipelines to process multimodal data and support near-real-time clinical and managerial decisions. Federated learning extends this adaptivity across institutions by allowing decentralised training without exchanging raw data, preserving privacy while enabling collective intelligence [46].

Data governance in integrated care must evolve from compliance to stewardship that enables responsible reuse, innovation, and equity. Emerging European debates on *digital personhood* and *algorithmic solidarity* clarify the same requirement: legitimate data reuse depends on demonstrating collective benefit while preserving individual agency, a process audited routinely by data-trusts and access bodies under frameworks like EHDS. Under these conditions, digital infrastructure can support continuous feedback between clinical operations and model performance. A practical governance checklist for Type V programmes thus includes: (i) clear roles for data controllers/processors and trusted intermediaries; (ii) consent and transparency mechanisms appropriate to longitudinal, multimodal analytics; (iii) model lifecycle obligations (drift monitoring, revalidation, versioned provenance, and explainability audits); (iv) equity safeguards that monitor and mitigate residual gaps across subgroups; and (v) reciprocal value frameworks so contributing sites and citizens see tangible benefit [25,29,35]. Embedding these expectations in contracts and oversight bodies reduces the uncertainty that often stalls cross-sector data collaboration and unlocks the learning benefits of scale. These governance conditions frame the broader synthesis taken up in the Discussion.

### Regulating Clinical AI: EU AI Act, MDR/IVDR, and FDA SaMD

The regulatory landscape for clinical AI matured substantially during the period covered by this review and now constrains the design space for Type IV and Type V systems in concrete ways. In the European Union, the Artificial Intelligence Act (Regulation (EU) 2024/1689) [182] classifies AI systems used as a safety component of a medical device, or as a Software as a Medical Device (SaMD) requiring notified-body conformity assessment under the Medical Device Regulation (MDR) or In Vitro Diagnostic Medical Device Regulation (IVDR), as high-risk by default; some additional categories (for example, emotion recognition or mental-health monitoring) are designated high-risk via Annex III [182,183]. The Medical Device Coordination Group’s MDCG 2025-6 makes explicit that the AI Act applies in parallel with the MDR/IVDR rather than supplanting it, addressing AI-specific hazards (drift, bias, opacity) that the device regulations do not [183]. The headline timing is that the full AI Act becomes applicable on 2 August 2026, with transitional provisions for devices already placed on the market before that date that do not undergo significant design changes [182]. In the United States, the Food and Drug Administration’s draft guidance on predetermined change control plans for AI/ML-enabled device software functions formalises a parallel idea: pre-specified, monitored change pathways are the regulatory route for adaptive AI in clinical use [184].

**Figure 8:**
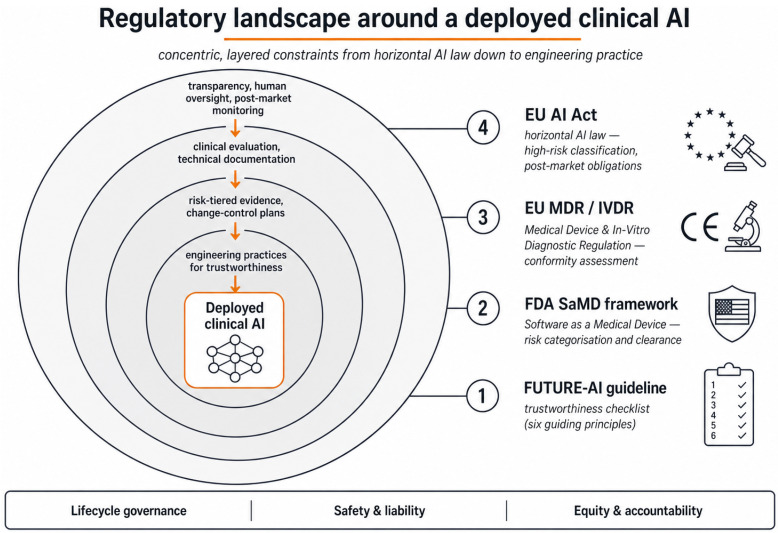
Regulatory and governance landscape around a deployed clinical AI system, drawn as four concentric layered constraints numbered from the innermost engineering-practice ring outward to the outermost horizontal AI law: (1) the FUTURE-AI consensus guideline as the innermost engineering-practice ring (a six-principle trustworthiness checklist spanning the AI lifecycle); (2) the FDA SaMD framework (risk-tiered evidence and predetermined change-control plans for adaptive AI in clinical use); (3) the EU MDR/IVDR (medical-device conformity assessment and clinical-evaluation requirements); and (4) the EU AI Act (horizontal AI law: high-risk classification, transparency, human oversight, and post-market monitoring obligations). The inward-pointing arrows label what each regime constrains in the design of a deployed model. The bottom band names the three governance dimensions that cut across all four regimes simultaneously: lifecycle governance, safety & liability, and equity & accountability. The figure is used in this section as a single reference for how a Type V architecture must be built with the regulatory regime in view from the start, rather than retrofitted as a downstream compliance task.

The integrated-care implication is concrete. A Type V programme cannot treat regulation as a downstream compliance task; it must architect the model lifecycle (drift monitoring, predetermined-change plans, fairness audits, post-market surveillance, traceable training-data lineage) for the regulatory regime in which it is deployed. The FUTURE-AI international consensus guideline—fairness, universality, traceability, usability, robustness, explainability across thirty best practices spanning the AI lifecycle—now provides a practical reporting and design vocabulary that operationalises both the AI Act and SaMD expectations [36]. The COMFORTage architecture, with explicit drift monitoring, XAI tooling (LIME/SHAP/DALEX) and FHIR-based provenance, is one example of designing the workflow with these regulatory expectations in view from the start; the more general lesson is that regulation is now a Type V design input, not a compliance afterthought. At project level, recent operationalisation efforts translate this guidance into actionable cyclical methodologies: the ETHAI methodology, for example, integrates EU Trustworthy AI principles with bioethics, care ethics and neuroethics through a four-phase loop of ethics-requirement identification, requirement translation, implementation and refinement, and assessment and evaluation, applied here in dementia and frailty care [24], mirroring on the ethics axis the same closed-loop logic that the stewarded learning loop carries on the model-lifecycle axis. Figure 8 summarises this layered regulatory and governance landscape around a deployed clinical AI system.

## 8. Discussion

The convergence of ICMs and AI adds a cognitive layer to integration. Predictive models, decision support, and feedback loops can anticipate need and coordinate action across settings. The central question is whether these capabilities are embedded in workflows that can learn over time. Two balances matter. First, human–AI symbiosis: AI should widen clinicians’ field of view and compress reaction time, not crowd out judgement. Second, temporal continuity: systems must recognise trajectories across months and years, not only episodes.

A unifying thread in this review is time. Type IV programmes wire data and deploy analytics, but they mostly observe snapshots. Type V systems treat *patient outcome trajectories*—frailty progression, cognitive decline, care-dependency transitions, and the response to interventions across years—as first-class objects. This shift changes both engineering and evaluation. Engineering priorities move from static dashboards to pipelines that detect drift, refresh models, and log provenance as populations and practices evolve. Evaluation moves from one-off accuracy to learning-cycle KPIs that are explicitly temporal: time from drift detection to validated redeployment; stability of recommendations across updates; sustained narrowing of fairness gaps; and, critically, lead-time gained before deterioration. The test of an integrated, intelligent model is whether it consistently bends trajectories towards better function and fewer crises.

Clinically, risk stratification and pathway optimisation reduce preventable deterioration and streamline team work. Organisationally, success hinges on governance, interoperability, and model stewardship. EHDS and GDPR foreground citizen rights and trustworthy reuse; within ICMs this implies accountable data stewardship, validation, and bias monitoring. Co-design with clinicians and patients is non-negotiable. So are incentives that reward prevention and longitudinal outcomes rather than volume.

Beyond routine EHRs, richer multimodalities are moving into scope for integrated care. Polygenic risk scores, multi-omics, and imaging biomarkers can complement clinical and behavioural signals to refine risk stratification and personalise prevention, provided they are interpreted within fair and transparent frameworks [185,186,187]. Operationally, cross-sector collaborations and government-academic data partnerships proved their worth in crises and should be institutionalised for routine improvement [179]. On the engineering side, dynamic embeddings and zero-shot sequence models show promise for generalising across institutions and tasks, but require strong governance to manage drift and explainability [188,189].

Ethically, AI can both support and undermine equity. Bias in training data, opaque models, and uneven access can widen gaps. Explainability, fairness audits, and privacy-preserving learning help; legitimacy ultimately rests on participation—citizens having a meaningful say in how data and algorithms shape their care. Equity itself has a temporal dimension: we should expect not only parity at a timepoint, but convergence of trajectories across groups when mitigation is working.

Evidence remains uneven. Many studies are pilots, with short follow-up and narrow metrics. Comparative, longitudinal evaluations and cost-effectiveness are scarce, especially in low- and middle-income countries (LMICs). Future work should use open-science practices and reporting standards (e.g., Transparent Reporting of a multivariable prediction model for Individual Prognosis Or Diagnosis, Artificial Intelligence (TRIPOD-AI) [190]; Developmental and Exploratory Clinical Investigation of Decision-support systems driven by Artificial Intelligence (DECIDE-AI) [191]; FUTURE-AI [36]), and prioritise pragmatic, multi-site studies that assess clinical, economic, equity, implementation, and workforce effects together. Crucially, studies should report temporal endpoints, lead-time to detection, durability of effect after model updates, and the resource required to maintain learning cycles. Dashboards age. Good studies measure how fast and what it takes to keep them honest.

**How this review sits alongside prior reviews.** Earlier syntheses of integrated care have tended to settle in one of two camps. The first camp documents organisational and policy integration with comparative depth but light digital treatment, exemplified by the Rainbow framework [49], the Chronic Care Model and its descendants [9], and global comparisons of integrated-care policy [157,158]. The second camp documents AI in clinical care with technical and benchmark depth but light integrated-care treatment, exemplified by reviews of clinical AI deployment [61,62], of federated learning in medicine [46], and of LLMs in clinical workflows [102,104,105]. Recent reviews that begin to bridge the two camps include Stoumpos et al.’s digital-transformation review of healthcare [192], Hwang et al.’s AI-in-integrated-care synthesis [33], Saberi et al.’s data-analytics-for-integrated-care review [30], the Krones et al. review of AI deployment in healthcare [78], and Mohammadi et al.’s 2026 scoping review of differential privacy in medical deep learning [181]; each contributes either a digital-transformation lens, an AI-in-coordination lens, or a privacy-and-deployment lens to the same intersection. The contribution of the present review is complementary: the Type I–V scheme treats organisational, payment, digital, and analytical integration as positions on the same axis rather than as separate literatures, benchmarks the scheme against the maturity models that the first camp has built (Rainbow, MacColl/CCM, EMRAM/AMAM, NHS ICS), and reads the second camp’s failure cases (Epic Sepsis, Watson for Oncology, COVID imaging models, LLM hallucination patterns) as evidence about what blocks progression from Type IV to Type V. What is new is the unified vocabulary and the explicit IV/V boundary, anchored in regionally balanced empirical examples spanning nine world regions.

### Limitations of This Review

Five limitations should temper the conclusions drawn here. First, this is a scoping review with a single primary reviewer who applied two-round co-author calibration. A fully independent dual-reviewer process across all 11,789 screened records was beyond the scope of the project. The principal risk is selection bias at the title-and-abstract stage, which we mitigated through an inclusive screening posture and by recording exclusion reasons at the full-text stage. Second, three of our four databases (Scopus, Crossref, Semantic Scholar) are biased towards Western and English-language indexing, which under-represents LMIC integrated-care work. This is flagged in Section 6.2, and LMIC examples are treated as illustrative rather than representative. Third, the Type I–V scheme is an analytical framework rather than a psychometrically validated instrument. Its primary value is as a charting tool and a vocabulary for the IV/V distinction, and the crosswalk in Table 4 should not be read as a formal validation. Fourth, the case vignettes (Section 6.1) are anchored in published material and project documentation. They do not include direct site visits, primary-source interviews, or quantitative within-case analysis, and the assignment to a Type level reflects a design-and-deployment reading rather than an audited maturity assessment. Fifth, we did not undertake a formal risk-of-bias appraisal of the empirical implementations summarised in Table S3 of the Supplementary Material. This is consistent with PRISMA-ScR guidance for scoping reviews [22] but limits the strength of the inferences that can be drawn from individual studies. Several of these limitations point directly to the next-step research priorities listed in Section 9, including dual-reviewer scoping or systematic reviews of specific Type V components and primary case studies with site engagement.

## 9. Future Directions

ICMs and AI are moving towards more adaptive learning systems. Several priorities are now clear: trustworthy and explainable (increasingly causal) AI to support accountable decisions, federated and privacy-preserving analytics for cross-institution collaboration, real-time data streams from wearables, home sensors, and community sources to enable adaptive closed-loop pathways, evaluation that values outcomes, experience, equity, and cost over accuracy alone, interoperable data spaces and shared ontologies (EHDS, Trusted Exchange Framework and Common Agreement (TEFCA) [193]) to stabilise semantics and governance, human–AI teaming with user experience (UX) and training that make the partnership usable in practice, explicit learning-cycle KPIs (latency from drift to redeployment, explainability compliance, fairness gap, model provenance), and workforce upskilling and co-design capacity so clinicians, data scientists, and citizens can shape solutions together. Together, these priorities place integration within a longer-term model of coordinated human and machine decision support.

Three pragmatic priorities emerge. First, invest in longitudinal data assets and semantics, since trajectory-aware phenotypes (frailty, care-dependency, cognition) and harmonised coding unlock earlier, fairer intervention. Second, standardise stewardship by monitoring drift, refreshing models, and reporting explainability and fairness routinely rather than episodically. Third, align incentives to longitudinal value, by using outcome-based contracts and shared-savings models that reward prevention and sustained equity improvement. Technically, continue advancing multimodal fusion and federated learning for privacy-preserving scale. Clinically, expand trials that evaluate impact on admissions, function, and experience. Organisationally, develop repeatable playbooks for government–academic–provider data collaboratives [46,47,179].

### A Practical Checklist for Type V Readiness

For implementers, funders, and reviewers, the following ten-item checklist consolidates the conditions that, in the corpus, repeatedly distinguish Type V designs from Type IV deployments masquerading as them. The checklist is descriptive, not prescriptive: it summarises the structural features that the empirical and conceptual literature converges on (Section 5, Section 6 and Section 7).

1. **Longitudinal multimodal data capture** (EHR, devices, social and environmental signals) integrated into a common longitudinal store, not held only as snapshots.
2. **Trajectory-aware phenotypes** (frailty, care-dependency, cognition) defined and computed routinely, not only at episodic touchpoints.
3. **Federated-by-default analytics** for cross-site learning under explicit data-space governance.
4. **Stewarded model lifecycle**: drift monitoring, scheduled retraining, versioned provenance, and a documented decommissioning pathway.
5. **Equity audit on every release**: stratified performance, fairness gaps, and mitigation, not only an aggregate AUC.
6. **Explainability fit-for-purpose**: rationales targeted at the actual decision and decision-maker, with measurable compliance.
7. **Outcome-based contracting** that pays for sustained outcomes rather than activity, creating a financial reason to maintain the loop.
8. **Workforce stewardship roles** (clinical-AI lead, model steward, data-trust officer) named in the operating model and resourced.
9. **Patient and carer co-design** embedded in the governance layer, not bolted onto launch communications.
10. **Learning-cycle KPIs reported alongside clinical KPIs**: time from drift detection to validated redeployment, explainability compliance, fairness gap, and model provenance.

A deployment that satisfies items 1–4 is operationally a Type V *in design*; one that satisfies all ten is a Type V *in practice*. Most deployments in the current corpus satisfy 0–3 of these and remain at Type IV; the gap between design intent and practice is itself the most useful unit of analysis for evaluators and funders.

## 10. Conclusions

Intelligent integrated care moves service delivery closer to learning systems organised around people, data, and longitudinal outcomes. Realising this promise means pairing technical progress with governance and participation so that models are safe, fair, and trusted. Integration must span providers, infrastructures, regulation, and citizen stewardship, with incentives aligned to prevention and longitudinal outcomes. When implemented with strong governance, AI can strengthen coordination, improve outcomes and experience, and support equity and sustainability. Poorly governed deployment risks reinforcing fragmentation, bias, and distrust. Progress therefore depends on co-design, rigorous evaluation, transparent governance, and workforce development.

Current programmes in digital primary care, integrated home care, patient-centred medical homes, rural cardiovascular management, and older-adult support show that the field is advancing, but mostly through context-specific Type IV configurations rather than mature Type V learning systems [45,114,115,116,117,118]. Early 2026 evidence strengthens this reading rather than overturning it. The field is adding stronger building blocks, transdisciplinary ageing frames, multimodal precision-health models, transitional-care orchestration, privacy-preserving architectures, and leadership-focused governance thinking, but these still mostly appear as partial Type V components rather than mature, governed learning systems [7,70,71,108,119,175,181]. The next step is not simply to add more algorithms. It is to pair longitudinal multimodal data with governed feedback loops, explicit model stewardship, and organisational arrangements that allow teams to learn safely over time. Under those conditions, integrated care can evolve from better-connected services into accountable learning systems for ageing populations and chronic disease management.

## Figures and Tables

**Table 1:** Inclusion and exclusion criteria applied at title/abstract and full-text stages.

Dimension	Inclusion	Exclusion
Topic	Conceptual definitions or frameworks of integrated care; digital or data infrastructures supporting integration; AI/ML or algorithmic components enabling ICM functions; empirical implementations or evaluations in health or social-care settings	Stand-alone technology evaluations with no integrated-care or coordinated-care framing; pure clinical-trial reports without an integration component; non-healthcare application domains
Study type	Conceptual or theoretical papers; narrative, scoping or systematic reviews; empirical implementation, evaluation, observational or trial studies; methods papers reporting ICM-relevant techniques; policy and grey literature with explicit provenance	Editorials, opinion pieces or commentaries with no analytical content; book reviews; conference abstracts without methods; predatory-journal items
Population and setting	Patients and care teams in primary, community, hospital, long-term, or social-care settings; population-level integrated-care programmes; policy or system-level analyses	Animal studies; *in vitro* or bench studies; consumer-only digital-health applications without a care-system anchor
Time and language	Records published January 2001–March 2026; English-language abstract or full text	Records published before 2001 unless cited as foundational anchors (e.g., the Chronic Care Model); items without retrievable abstract or full text
Provenance	Indexed in PubMed, Scopus, Semantic Scholar, or Crossref; or grey literature from named bodies (WHO, OECD, European Commission, national health agencies); or foundational items from the project reference library cross-checked against the search	Records with no traceable bibliographic identifier; duplicate publications of the same study

**Table 2:** Records identified per database and per conceptual tier (search retrieval date 31 October 2025; complemented by targeted forward screening to 31 March 2026). Counts are reproducible from the per-database RIS exports archived with the Supplementary Materials.

Database	Tier 1 (AI)	Tier 2 (ICM)	Tier 3 (Trans.)	Raw Total	Intra-Source Unique
PubMed	599	399	21	1019	1018
Scopus	200	200	45	445	445
Crossref	1000	1000	1000	3000	2709
Semantic Scholar	5000	5000	725	10,725	7978
Total raw	6799	6599	1791	15,189	12,150
After cross-database deduplication (DOI-first, normalised-title fallback)					11,744
Plus grey literature and project reference library cross-check					11,789

**Table 3:** Proposed taxonomy of Integrated Care Models (Type I–V), with integration focus, coordination mechanisms, digital and AI maturity, governance arrangement, and illustrative examples. Each row corresponds to one rung of the stepped ladder shown in Figure 2, and representative citations anchor each Type in the empirical and policy literature.

ICM Type	Integration Focus	Typical Coordination Mechanisms	Digital/AI Maturity Level	Governance Model	Illustrative Examples (with Representative Citations)
Type I: Disease-Specific Programmes	Vertical integration around a single disease or condition.	Clinical guidelines, disease registries, shared protocols.	Level 1: EHR or registry-based systems, minimal analytics.	Project-based or siloed management.	Diabetes networks, oncology pathways [9,38].
Type II: Population-Segmented ICMs	Integration for defined cohorts such as older adults, frail or multimorbid patients.	Case management, multidisciplinary teams, transitional care programmes.	Level 2: interoperable data exchange between providers.	Networked governance across primary and community care.	Frailty programmes, chronic-care networks [39,40].
Type III: Whole-System ICMs	System-wide integration across hospital, primary, and social care.	Joint commissioning, shared budgets, population-health management.	Level 3: predictive analytics and population dashboards.	Federated or regional governance.	Regional accountable-care organisations, NHS Integrated Care Systems [41,42,43].
Type IV: Digital-Integrated ICMs	Integration driven by digital platforms, remote monitoring, and interoperability.	Shared data hubs, telehealth services, real-time communication tools.	Level 4: AI-assisted decision support, early-warning systems, automation.	Federated governance with algorithmic oversight.	Smart-hospital networks, digital-health platforms, EHDS pilots [19,44,45].
Type V: Learning ICMs	Continuous, adaptive integration through AI/ML feedback loops and data spaces.	Federated AI, reinforcement-learning systems, outcome-based contracting.	Level 5: learning health systems with real-time model retraining and fairness auditing.	Adaptive, data-driven governance structures.	Population-health systems with embedded AI, predictive ageing-care platforms [46,47,48].

Levels 1–5 denote increasing digital and AI maturity from EHR coordination to learning health systems. Coordination mechanisms, governance arrangements, and illustrative examples are indicative rather than exhaustive, and the same implementation may sit between two adjacent Types in practice.

**Table 4:** Crosswalk between the Type I–V scheme used in this review and selected existing integrated-care, chronic-care, and digital-maturity frameworks. Cells indicate the closest comparable construct in each external framework; not every external framework has an equivalent at every Type level.

This Review	Rainbow Model [49]	Development Model [51]	Chronic Care Model [9]	HIMSS EMRAM/AMAM [52,53]	NHS ICS/WHO GSDH [54,55]
Type I (disease)	Clinical micro-integration	Initiative phase, single condition cluster	CCM applied to one disease pathway	EMRAM 0–2 (paper or basic EHR)	Pre-formal integration; basic digital readiness
Type II (cohort)	Clinical + professional integration for a defined population	Experimentation phase across professionals	CCM applied across cohort; care managers	EMRAM 3–4 with limited analytics	Population-segment focus; emerging shared services
Type III (whole-system)	Organisational + system integration	Expansion and consolidation across organisations	CCM at system scale; community linkages	EMRAM 5–6 with population-health reporting	“Maturing” ICS; national strategy in place
Type IV (digital-integrated)	Functional integration through ICT and shared records	Sustainability phase with shared digital platform	CCM with mature information systems and decision support	EMRAM 6–7; AMAM 4–6 (analytics in workflow)	“Thriving” ICS; advanced national digital infrastructure
Type V (learning)	Not explicitly addressed; closest is normative integration with adaptive governance	Not explicitly addressed; would extend phase 4 with continuous improvement of digital + analytical layers	Not explicitly addressed; CCM is a service-design model	AMAM 7–8 (predictive and prescriptive analytics in routine use) extended with stewarded model lifecycle	WHO GSDH “governed digital ecosystems” axis; no equivalent in NHS ICS matrix

**Table 5:** Worked classification of three implementations against the five coding dimensions used during charting. The three cases span the Type IV/V boundary.

Implementation	Unit of Integration	Coordination Mechanism	Digital & Analytics Maturity	Governance Arrangement	Learning Loop	Type
NHS Federated Data Platform [57]	Whole-system, region-spanning	Shared data hub + regional governance forums	Federated EHR access; descriptive and predictive analytics; AI-assisted operational tools	Federated, with national algorithmic-oversight expectations	Periodic model refresh; no closed-loop deployment yet	IV
Geisinger ProvenCare and population-health programmes [58,59]	Whole-system, vertically integrated payer-provider	Shared budgets; protocol bundles; population-health management	Mature EHR; predictive risk stratification in workflows; AMAM-equivalent of 6–7	Federated organisational governance with outcome-based contracts (ProvenCare)	Routine model retraining for risk scores; partial closed loop tied to ProvenCare warranty	IV/V
COMFORTage Integrated Care Model Library inside the Virtual Health Platform [37,60]	Cross-country ageing ecosystem; ICM lifecycle from prevention to follow-up	Workflow of AI agents orchestrating predictive, preventive, and personalised care across sites; explicit data-space governance; outcome-based co-design	Multimodal data fusion (clinical, behavioural, wearable); automated model selection; continuous learning with drift monitoring; explainable AI (LIME, SHAP, DALEX); Holistic Health Records over FHIR with blockchain-backed audit	Adaptive data-driven governance through a data-space and trusted intermediary, aligned with Trustworthy AI guidance	Workflow-level model retraining and stewardship across the ICM lifecycle; explicit Type V design intent on the agentic-orchestration axis	IV with V intent

**Table 6:** Why Type IV fails and how Type V responds in Integrated Care Models.

Failure in Type IV	Typical Symptom	Underlying Cause	Type V Response
Temporal blind spots [10,72,113]	Late detection of decline and reactive escalations	Snapshot metrics and episode-centred data	Model for life-course trajectories and early-warning signals from multimodal streams
Maintenance debt [30,33]	Models drift and dashboards become untrusted	No resourced retraining or revalidation	Stewarded ML lifecycle: drift monitoring, scheduled retraining, versioned provenance
Semantic drift [10,120]	Handoffs fail and shared plans diverge	Inconsistent codes/ontologies across sites	Shared ontologies, semantic mediation, and governance for controlled vocabularies
Governance gaps [19,46]	Privacy/security block reuse and stalled collaborations	Compliance-only mindset, unclear roles	Data-trusts and data-spaces with clear access, audit, and shared benefit rules
Appraisal mismatch [10,33]	High AUC (area under the ROC curve), low impact	Metrics ignore workflow fit and equity	Learning KPIs (latency, explainability compliance, fairness gap) alongside outcomes
Engagement as app [10,12]	Low uptake and inequalities widen	Tools without support for agency	Co-production, assisted navigation, and equity monitoring with mitigations

## Data Availability

This manuscript does not report data generation or analysis.

## Supplementary Materials

The following supporting information can be downloaded at: https://www.mdpi.com/article/10.3390/healthcare14121612/s1. Section S1: PRISMA-ScR Checklist, including Table S1 (22-item PRISMA-ScR checklist mapped to manuscript locations). Section S2: Supplementary Methods and Extended AI Materials, including Table S2 (per-database identification counts), Figure S1 (PRISMA-style audit trail of the search and selection process), the verbatim Tier 1–3 Boolean search blocks, the screening workflow and inter-rater agreement, search-recall validation, the eligibility, appraisal, data-extraction, and taxonomy-coding rules, and six extended-AI subsections supporting Section 4 of the main manuscript. Section S3: Empirical Implementations Foregrounded in the Synthesis, including Table S3 (full 40-entry reference table of empirical implementations across nine world regions and the Type I–V range, rendered in landscape orientation, and summarised narratively in Section 6.3 of the main manuscript). Section S4: Supplementary Policy Frameworks (additional policy instruments beyond the three anchor frameworks referenced in the Introduction). The full Type I–V taxonomy with dimensions and illustrative examples is now reported in the main manuscript as Table 3. The per-database, per-tier RIS exports underlying the search counts in Section 3.1 of the main manuscript are archived alongside this supplementary file in raw_search_exports/. The additional implementation and policy sources drawn on only in the Supplementary Material are also included in the reference list of this manuscript [194,195,196,197,198,199,200,201,202,203,204,205,206,207].

## Abbreviations

The following abbreviations are used in this manuscript.

AI	Artificial Intelligence
AMAM	Adoption Model for Analytics Maturity (HIMSS)
API	Application Programming Interface
AUC	Area Under the ROC Curve
CCM	Chronic Care Model
CCMC	Compact County Medical Alliance (China)
CDM	Chronic Disease Management
CDS	Clinical Decision Support
CPM	Care Process Model (Intermountain)
DALEX	Descriptive mAchine Learning EXplanations
DECIDE-AI	Developmental and Exploratory Clinical Investigation of Decision-support systems driven by Artificial Intelligence
DIPH	Data-Informed Platform for Health (Ethiopia)
DL	Deep Learning
EBAIS	Equipo Básico de Atención Integral en Salud (Costa Rica)
ED	Emergency Department
EHDS	European Health Data Space
EHR	Electronic Health Record
EMRAM	Electronic Medical Record Adoption Model (HIMSS)
ETHAI	Embedding Ethics in Healthcare AI (project methodology)
EU	European Union
FDA	U.S. Food and Drug Administration
FHIR	Fast Healthcare Interoperability Resources
FUTURE-AI	International consensus guideline for trustworthy AI in healthcare
GDPR	General Data Protection Regulation
GSDH	Global Strategy on Digital Health (WHO)
HIMSS	Healthcare Information and Management Systems Society
HL7	Health Level Seven
HTA	Health Technology Assessment
ICM	Integrated Care Model
ICML	Integrated Care Model Library (COMFORTage)
ICS	Integrated Care System (NHS)
IoT	Internet of Things
IPCHS	Integrated, People-Centred Health Services
IVDR	In Vitro Diagnostic Regulation (EU)
KPI	Key Performance Indicator
KPIN	Kaiser Permanente Intelligent Navigator
LIME	Local Interpretable Model-agnostic Explanations
LLM	Large Language Model
LMIC	Low- and Middle-Income Country
MDR	Medical Device Regulation (EU)
MDT	Multidisciplinary Team
ML	Machine Learning
NCD	Non-Communicable Disease
NHS	National Health Service
NLP	Natural Language Processing
OECD	Organisation for Economic Co-operation and Development
PHC	Primary Health Care
PM-JAY	Pradhan Mantri Jan Arogya Yojana (India)
PREM	Patient-Reported Experience Measure
PRISMA-ScR	Preferred Reporting Items for Systematic Reviews and Meta-Analyses, extension for Scoping Reviews
PROM	Patient-Reported Outcome Measure
RIS	Research Information Systems (citation export format)
ROI	Return on Investment
SaMD	Software as a Medical Device
SHAP	SHapley Additive exPlanations
SNOMED CT	Systematized Nomenclature of Medicine Clinical Terms
TEFCA	Trusted Exchange Framework and Common Agreement
TRIPOD-AI	Transparent Reporting of a multivariable prediction model for Individual Prognosis Or Diagnosis, Artificial Intelligence
UHC	Universal Health Coverage
UX	User Experience
WHO	World Health Organization
XAI	Explainable Artificial Intelligence
