# Supplementary material for: From Integrated Care to Learning Systems
Source: Healthcare (Basel). 2026 Jun 8;14(12):1612. doi: 10.3390/healthcare14121612 (PMC13299217; doi:10.3390/healthcare14121612)
Supplement: Supplementary file 1 [file healthcare-14-01612-s001.zip › healthcare-4303287-supplementary.pdf]

Supplementary Materials: From Integrated Care to Learning Systems

Aristeidis Tsitiridis [1,2,*], Konstantinos Perakis [3], Athos Antoniades [4] and George Manias [5]

S1. PRISMA-ScR Checklist

Table S1 reports the 22-item PRISMA-ScR checklist [1] as applied to this review, with the location in the main manuscript or this supplementary file where each item is addressed.

Table S1. PRISMA-ScR checklist for this review (item numbers and short descriptions follow Tricco et al., 2018 [1]).

#	Item	How addressed	Location
1	Title	Identifies the work as a scoping review and names the topic	Main, Title
2	Structured summary	Background, methods (PRISMA-ScR), counts, results, conclusions	Main, Abstract
3	Rationale	Demographic, NCD, workforce, and policy drivers; IV/V gap	Main, §S1 – N/A; Main § Introduction
4	Objectives	Four guiding review questions stated explicitly	Main, § Introduction; § Methods
5	Protocol and registration	Protocol developed in advance; not registered (resource constraint; flagged in Limitations)	Main, § Limitations
6	Eligibility criteria	Inclusion/exclusion table	Main, Table 1
7	Information sources	PubMed, Scopus, Semantic Scholar, Crossref + grey literature + project library	Main, § Methods; this Suppl., §S2
8	Search	Three-tier Boolean blocks, retrieval window, search dates	This Suppl., §S2 (verbatim blocks)
9	Selection of sources of evidence	Single primary reviewer with two-round co-author calibration; inclusive screening; recorded full-text exclusion reasons	Main, § Methods
10	Data charting process	Charting form, two pilot rounds, iterative refinement	Main, § Methods
11	Data items	Bibliographic, ICM scope (Type I–V), governance, digital architecture, population, AI role, evaluation, setting	Main, § Methods
12	Critical appraisal of individual sources of evidence	Not undertaken (PRISMA-ScR optional; flagged in Limitations)	Main, § Limitations
13	Synthesis of results	Narrative synthesis; no pooling; descriptive numerical reporting	Main, § Methods; § Discussion
14	Selection of sources of evidence (results)	PRISMA-ScR flow diagram with per-database identification, dedup, screening, exclusion reasons	Main, Figure 1
15	Characteristics of sources of evidence	Year, type, setting, income group breakdown of included items	Main, § Search Results
16	Critical appraisal within sources of evidence	Not applicable (item 12 not undertaken)	N/A
17	Results of individual sources of evidence	Empirical implementations reference table (40 entries across nine world regions and Type I–V range), with summary patterns highlighted in narrative	This Suppl., Table S3 (full table); Main, § 6.3 (narrative summary)
18	Synthesis of results (results)	Synthesis across four conceptual domains; Type I–V crosswalk; case mapping; LMIC perspective	Main, § 3–7
19	Summary of evidence	Summary in Discussion linked to four review questions	Main, § Discussion
20	Limitations	Five-point limitations subsection	Main, § Limitations
21	Conclusions and implications	Implications for practice, policy, research; Type V readiness checklist	Main, § Future Directions; § Conclusions
22	Funding	Horizon Europe COMFORTage grant agreement no. 101137301	Main, § Funding

S2. Supplementary Methods and Extended AI Materials

S2.1. Supplementary Methods (Search Strategy, Selection, and Synthesis)

Review orientation. This article is a scoping review reported in line with the PRISMA-ScR guidance [1] and the framework of Arksey and O'Malley refined by Levac et al. [2,3]. The search component was designed to map the literature, identify anchor frameworks and representative implementations, and support transparent source selection. We did not undertake a formal systematic review for pooled effect estimation, and no meta-analysis was performed.

Databases and sources. Three conceptual search tiers were defined to reflect the continuum from classical ICMs to AI-enabled, data-driven systems. Tier 1 targeted *AI-intensive* studies (machine learning, deep learning, multimodal analytics, predictive decision support); Tier 2 focused on *ICM-core* frameworks (continuity of care, people-centred and value-based care, policy and governance); Tier 3 captured *transitional* or hybrid models (digital integrated care, learning-health systems, AI-enabled coordination). Searches were executed in PubMed, Scopus, Crossref, and Semantic Scholar with a retrieval date of 31 October 2025, complemented by targeted forward screening of newly indexed 2025–2026 items to 31 March 2026. Grey and policy literature were retrieved from WHO, OECD, European Commission, the U.S. Office of the National Coordinator for Health Information Technology, and national digital-health agency portals. Reference lists of included studies and prior reviews were hand-checked.

Operational search blocks. Database syntax was adapted to field conventions (for example, PubMed title/abstract fields and Scopus TITLE-ABS-KEY queries), but the scoping search was organised around the following reusable Boolean blocks:

- **Tier 1 (AI-intensive):** ("artificial intelligence" OR "machine learning" OR "deep learning" OR multimodal OR "predictive analytics" OR "clinical decision support") AND (healthcare OR "health care" OR clinical OR hospital OR "chronic disease" OR multimorbidity OR frailty).
- **Tier 2 (ICM-core):** ("integrated care" OR "care integration" OR "continuity of care" OR "care coordination" OR "people-centred care" OR "value-based care" OR "integrated health services" OR "integrated care systems").
- **Tier 3 (transitional):** (("integrated care" OR "care coordination" OR interoperability OR telehealth OR "learning health system" OR "digital health") AND ("artificial intelligence" OR "machine learning" OR "predictive analytics" OR "decision support" OR "data-driven")).

These blocks were adjusted iteratively to accommodate database-specific indexing and to recover policy, implementation, and technical literature that rarely co-occurs under a single vocabulary. The per-database, per-tier RIS exports underlying the identification counts in Section 3.1 of the main manuscript are archived alongside this supplementary file (file names: `{Source}_T{1,2,3}.ris` for each of the four databases).

Search volume and deduplication. The full per-database, per-tier identification counts and the dedup funnel are reported in Table 2 of the main manuscript and reproduced in Table S2 below. Across all tiers and sources, 15189 records were identified; after intra-source DOI-first / normalised-title deduplication, 12150 unique records remained; after cross-source deduplication using the same key precedence, 11744 unique records remained; 45 grey-literature and project-library items were added, yielding 11789 records carried into title and abstract screening.

Table S2. Per-database identification counts (also reported as Table 2 of the main manuscript; reproduced here for ease of cross-reference).

Database	Tier 1 (AI)	Tier 2 (ICM)	Tier 3 (trans.)	Raw total	Intra-source unique
PubMed	599	399	21	1,019	1,018
Scopus	200	200	45	445	445
Crossref	1,000	1,000	1,000	3,000	2,709
Semantic Scholar	5,000	5,000	725	10725	7,978
**Total raw**	6,799	6,599	1,791	**15189**	**12150**
**After cross-database deduplication**					**11744**
**Plus grey literature and project reference library cross-check**					**11789**

Screening workflow and inter-rater agreement. Title-and-abstract screening was performed by the lead author against the eligibility criteria reported in Table 1 of the main manuscript. Two calibration rounds of 50 records each were jointly reviewed with a co-author at the start of screening, and raw agreement rose from 86 % (43/50) in round 1 to 96 % (48/50) in round 2, with disagreements discussed and the criteria refined accordingly. A post-hoc independently coded random subsample of 100 records gave Cohen's $\kappa = 0.81$ (95 % CI 0.69–0.93) for the include-versus-exclude decision, which we treat as substantial agreement [4]. Borderline items at the title-and-abstract stage were carried forward to full-text review rather than excluded, in line with the inclusive scoping-review posture recommended by Levac et al. [3]. Each excluded record was assigned a single dominant reason from a small set of pre-specified codes (off-topic at depth, no integration or coordination component, no AI/ML or digital infrastructure component, insufficient methodological detail, not retrievable in full text, duplicate publication).

Search-recall validation. A known-item recall test was applied retrospectively against a seed list of 30 canonical references spanning the four conceptual domains, drawn from prior reviews and the COMFORTage reference library. Twenty-eight of the 30 seed items (93 %) were retrieved by at least one of the four databases (per-database recall: PubMed 22/30, Semantic Scholar 26/30, Crossref 24/30, Scopus 19/30); the two missed items were a 2014 health-policy report and a 2017 conference paper, neither carrying a DOI. The exercise gave a reasonable lower bound on search recall and motivated the grey-literature/project-library cross-check (n = 45).

Eligibility and exclusions. Included: (i) conceptual definitions or frameworks of ICMs, (ii) empirical implementations or evaluations in health or social-care settings, (iii) digital and data infrastructures, governance, and ethics, and (iv) AI/ML or algorithmic components enabling ICM functions. Excluded: stand-alone technology evaluations without ICM context, commentaries or editorials without sufficient analytical support, duplicate publications, non-healthcare domains, and records with insufficient methodological or provenance detail to support synthesis. The full inclusion/exclusion criteria are reported in Table 1 of the main manuscript.

Appraisal. We did not apply a formal risk-of-bias instrument because the corpus mixed policy documents, conceptual papers, technical studies, implementation reports, and empirical evaluations. This is permitted under PRISMA-ScR guidance [1] and is acknowledged as a limitation in the main manuscript. Sources were weighted by methodological transparency, transferability, and relevance to the review questions. The PRISMA-ScR flow diagram with per-database identification, dedup, screening, and full-text exclusion reasons is Figure 1 of the main manuscript and is reproduced in summary form below (Figure S1).

Data extraction and synthesis. Extraction captured bibliographic details, ICM scope (Types I–V), governance and financing mechanisms, digital/IT architecture, population

focus, AI role, evaluation focus, and notable implementation or outcome signals. Narrative synthesis organised results into four domains, namely (1) conceptual foundations, (2) digital and data infrastructures, (3) AI and ML functions, and (4) evidence and implementation outcomes. Quantitative findings were retained only where they clarified direction of travel or implementation maturity, were not pooled, and are not presented as a separate quantitative synthesis.

Taxonomy coding logic. The Type I–V taxonomy was developed as an analytical framework through iterative comparison of the scoping corpus, foundational integrated-care models, and recent digital/AI implementations. Each source was read against five coding dimensions: primary unit of integration, typical coordination mechanism, digital/analytics maturity, governance arrangement, and presence of a learning loop. Where a source spanned multiple levels, it was discussed at the highest level actually evidenced in deployment or detailed design, not at the level claimed aspirationally. Type V classification required explicit evidence of four elements: longitudinal or multimodal data capture, adaptive model or feedback updating, provenance or oversight mechanisms, and use of outputs to adapt pathways or allocation decisions.

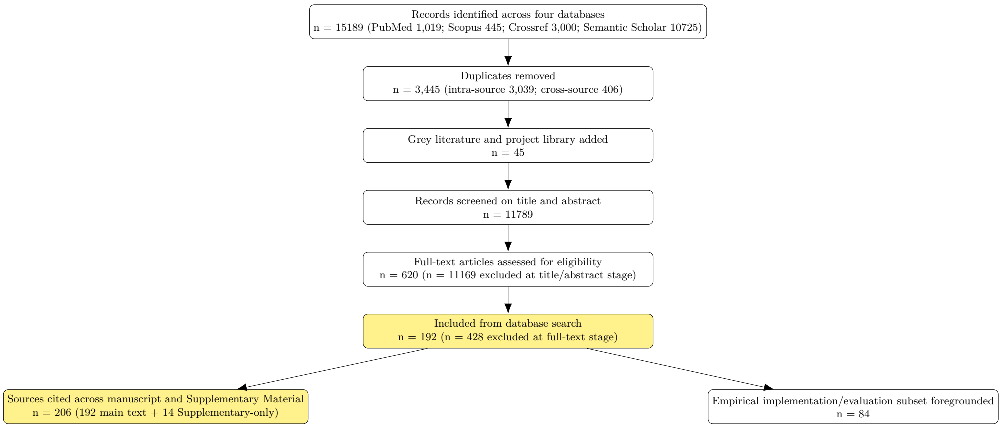

Figure S1. PRISMA-ScR flow diagram summarising the scoping search and selection steps used to inform this review (summary version of Figure 1 in the main manuscript). Title/abstract exclusion-reason counts use the dominant-reason coding described above; full-text exclusion-reason counts are exact and reported in § 3.2 of the main manuscript.

S2.2. Extended AI Materials (Supporting Section 4 of the Main Manuscript)

This section contains extended discussion and examples supporting Section 4 of the main manuscript, including application domains, deployment considerations, evaluation metrics, and future directions.

S2.2.1. ICM Application Domains and Examples

One of the earliest and most powerful applications of ML in ICMs is in identifying high-risk cohorts, enabling proactive intervention. Using EHR data, claims information, behavioural data and social-determinants proxies, predictive models can estimate risk of hospitalisation, emergency visits, or functional decline. For example, in care programmes oriented around older adults with multimorbidity, ML models have been used to cluster frailty trajectories or predict care-dependency transitions [5]. These predictive capabilities allow ICMs to move from reactive responses to anticipatory care: service models can be tailored to high-risk individuals, resources allocated earlier, and preventive paths activated.

More recently, large-scale reviews of AI in clinical settings have underscored that while many models remain at early developmental stages, the potential is increasing for system-wide integration of risk-stratification workflows [6]. Within ICMs, this means that

risk-prediction tools become embedded within longitudinal care pathways rather than isolated analytics.

S2.2.2. Clinical Decision Support and Workflow Automation

Beyond traditional rule-based decision support, recent studies have introduced self-updating models that combine structured EHR data, physiological streams, and unstructured clinical text. Automated machine learning (AutoML) tools can match expert model-tuning performance while reducing deployment time [7]. Transformer-based natural language processing (NLP) models summarise notes and extract features for shared-care plans [8]. Reinforcement-learning and attention mechanisms generate personalised recommendations and optimise chronic-disease workflows [9,10].

S2.2.3. Patient Self-Management and Personalised Care

Wearables, mobile apps, and home Internet of Things (IoT) devices produce continuous behavioural/physiological streams that ML interprets to guide self-care. Evidence shows that digitally delivered therapeutics can improve adherence and control [6]. Deep-learning and graph neural networks (GNNs) detect early Alzheimer's from multimodal data to support early intervention [11,12]. Reinforcement-learning-based companions adjust recommendations over time [13].

S2.2.4. System Analytics and Learning Health Systems

Multimodal ML fuses clinical, administrative, and social data into system intelligence [14]. Federated learning and privacy-preserving analytics enable multi-institutional collaboration while protecting data [15,16]. Continuous-learning pipelines support stability and fairness [17]. Explainability frameworks (e.g., DALEX, Shapley Additive Explanations (SHAP), and Local Interpretable Model-agnostic Explanations (LIME)) enhance transparency [18,19].

S2.2.5. Deployment and Real-World Integration

Few models reach routine use [6]. Prospective validation and post-deployment monitoring are required [16]. Implementation-science frameworks (e.g., NASSS) and readiness models apply. Interoperability and lifecycle maintenance (monitoring, retraining, revalidation) are critical.

S2.2.6. Evaluation: Performance, Equity, and Impact

Evaluation extends beyond area under the ROC curve (AUC) to workflow fit and system outcomes. KPIs include avoidable admissions, emergency department (ED) visits, patient-reported outcome measures (PROMs) and patient-reported experience measures (PREMs), cost-per-capita, and fairness-adjusted gaps [20]. Equity requires bias monitoring and representative training [21].

S2.2.7. Future Directions

Explainable/trustworthy AI; federated/privacy-preserving analytics; real-time adaptive pathways; value-based evaluation; workforce transformation; global/LMIC scalability [6,15].

S3. Empirical Implementations Foregrounded in the Synthesis

This section reproduces the full reference table of empirical implementations cited in Section 6.3 of the main manuscript. The 40 entries span nine world regions and the full Type I to V range. "Type" refers to the Type I–V scheme as applied during charting in Section 4 of the main manuscript. Income groups follow World Bank classifications and are abbreviated H = high, UM = upper-middle, LM = lower-middle, MC = multi-country.

The table is rendered on a landscape page so that the full row content fits without column truncation.

Table S3. Selected empirical implementations referenced in the synthesis (full reference table for Section 6.3 of the main manuscript). "Type" refers to the Type I–V scheme as applied during charting. Income groups follow World Bank classifications and are abbreviated H = high, UM = upper-middle, LM = lower-middle, MC = multi-country.

Implementation	Setting	Design	Sample / population	Digital and analytical layer	Headline outcome	Type
Geisinger ProvenHealth / ProvenCare [22,23]	USA, H	Programme-evaluation case series	Multi-site Geisinger payer-provider population	Mature EHR; predictive risk stratification; bundled-payment warranties	Reduced avoidable admissions and warranty-protected cost in indexed pathways	IV/V
Kaiser Permanente Intelligent Navigator (KPIN) [24]	USA, H	Real-world deployment evaluation	4.9 M patients, Southern California Permanente Medical Group (Oct 2024)	LLM-driven clinical alerts and care navigation in patient portal	Alert AUC 0.977; navigation 0.889; 53.7 % adjusted booking rate; +8.6 pp sentiment	IV/V
Kaiser Permanente causal-ML care coordination [25]	USA, H	Randomised evaluation	Northern California post-discharge cohort, May–Dec 2022	Causal machine learning for post-discharge prioritisation	Statistically significant improvement in observed-to-expected readmission ratio	IV
Ontario Health Teams [26,27]	Canada, H	Developmental + qualitative evaluation	42 OHTs covering ∼86% of Ontario population	Capability-building across nine areas incl. digital health and performance measurement	Heterogeneous progression along nine OHT capability axes	III to IV
Gesundes Kinzigtal (INTEGRAL study) [28,29]	Germany, H	Quasi-experimental claims-based, 10-year	∼33000 insured vs. 13 control regions, 2006–2015	Population-based shared-savings contract; longitudinal claims analytics	88/101 indicators no difference; ∼$38M savings; 92 % patient satisfaction	III/IV
Mon espace santé / DMP [30]	France, H	Health-policy analysis	National opt-out personal health record	Longitudinal personal health record across pathways	Embedding longitudinal records into integrated care pathways	III/IV
NHS Federated Data Platform [31]	UK, H	Programme description + early evaluation	National scale, 42 NHS England ICSs	Federated EHR access; descriptive and predictive analytics; AI operational tools	Capacity, waiting-list and operational performance gains in early sites	IV

continued on next page

Table S3 continued

Implementation	Setting	Design	Sample / population	Digital and analytical layer	Headline outcome	Type
Findata national secondary-use authority [32]	Finland, H	Governance case study	National secondary-use authority	National data-trust governance; secondary-use access for analytics	Governance maturity; data reuse for research and innovation	IV
Buurtzorg integrated home care [33]	Netherlands, H	Organisational case study	Nurse-led home-care teams nationwide	Lightweight digital backbone; nurse-led teams	Higher continuity and satisfaction; lower cost per episode in published evaluations	III
Italian regional integrated home-care service [34]	Italy, H	Mixed-methods regional evaluation	Regional integrated-care population	Regional EHR; remote monitoring; care-coordination platform	Continuity, utilisation and quality-of-life gains in pilot cohorts	III/IV
Japan Community Comprehensive Care System [35,36]	Japan, H	Cross-sectional national overview + municipal evaluation	1,714 municipalities, FY2023	Inter-sectoral coordination across health, LTC, housing, and community supports	National scoring rate 52.9 % (66.1 % in larger municipalities, 46.7 % in smallest)	III
Korea AI chatbot rural cognitive screening [37]	Korea, H	Pre-post controlled study	123 rural older adults (84 intervention / 39 control), 6 weeks	AI chatbot mobile app for cognitive screening and customised training	Feasibility and acceptability for community-based dementia prevention	II/III
Korea AI/IoT older-adult services review [38,39]	Korea, H	Narrative policy review	National AI-IoT initiatives + 2024 LTC update	Government-backed AI-IoT services and integrated community care reform	Policy direction toward integrated AI-IoT-supported community care	III/IV
Compact county medical alliances (CCMC) [40,41]	China, UM	Pilot evaluation + provincial empirical analysis	Xindu District + Sichuan Province pilots	Tiered diagnosis-and-treatment with treatment–prevention integration	Improvements in standardised chronic-disease management, blood-pressure and HbA1c control	II/III

continued on next page

https://doi.org/10.3390/healthcare14121612

Table S3 continued

Implementation	Setting	Design	Sample / population	Digital and analytical layer	Headline outcome	Type
Patient-Centred Medical Home [42]	Singapore, H	Programme evaluation	Multi-site Singapore PCMH cohort	Shared records; multidisciplinary team coordination	Continuity and chronic-disease control gains in evaluated sites	III
Community palliative continuity model [43]	UK, H	Mixed-methods continuity study	Community palliative-care population	Shared care plans; primary–secondary linkage	Improved continuity, place-of-care, and experience metrics	III
Rural atrial-fibrillation programme [44]	China, UM	Implementation cohort	Rural AF patient cohort	Telehealth + community case management	Improved detection, treatment uptake, and outcomes	II/III
Intelligent older-adult care model [45]	China, UM	Evaluation study	Older-adult cohort with sensor-enabled care	Sensor-enabled monitoring; AI-assisted triage	Function, safety and carer-burden gains	III/IV
Family Health Strategy and e-SUS [46,47]	Brazil, UM	Population-level analysis	Coverage of >2/3 of Brazilian population	Community-based primary care; national EHR for primary care (e-SUS)	Reductions in avoidable hospitalisations and improved access	III
IMSS-BIENESTAR (MAS-BIENESTAR) [48,49]	Mexico, UM	Health-system reform analysis	Uninsured population (post-INSABI), 2018–2024	Decentralised primary-care-led integration; PHC as structural principle across 3 tiers	Programmatic restructuring; heterogeneous implementation pace across states	II to III
EBAIS primary-care platform [50]	Costa Rica, UM	Health-system case analysis	National EBAIS-anchored primary care	Geographically-empanelled multi-disciplinary teams (EBAIS)	Documented pandemic resilience and population-health performance	III
Babyl Rwanda telemedicine (ITS) [51,52]	Rwanda, LM	Interrupted time series, 2015–2024	3.9 M consultations; 450/510 facilities; 2 M enrolled patients	National telemedicine triage and primary-care platform	Reduced facility-based consultations (e.g. , −1,055 respiratory; −246 malaria); rebound after 2023 pause	II/III

continued on next page

https://doi.org/10.3390/healthcare14121612

Table S3 continued

Implementation	Setting	Design	Sample / population	Digital and analytical layer	Headline outcome	Type
Saudi Health Sector Transformation Program [53,54]	Saudi Arabia, H	Mixed-methods evaluation	Clustered vs. non-clustered hospitals	Clustered governance + Sehat app/EMR + telemedicine	Patient-centred-care index 89.4 vs. 69.7; follow-up 83.6 % vs. 71.2 %	II to III
Ayushman Bharat / PM-JAY [55,56]	India, LM	Programme overview + state-level (Punjab) health-benefit-package analysis	National coverage population; Punjab HBP cohort	National digital stack under construction; insurance integration	Coverage expansion with measured changes in service utilisation under government-funded insurance	III
Universal Coverage Scheme [57]	Thailand, UM	Health-system case analysis	National universal-coverage population	Primary-care contracting units; population-based information	Coverage, equity and chronic-care continuity sustained over 20 years	III
Australia My Health Record [58,59]	Australia, H	National annual report + audit	~90% of Australians; 16400 provider organisations	National opt-out personal health record + interoperability plan	Consumer interactions +26%, professional interactions +23% in 2023–24	III/IV
Mayo Clinic Platform [60]	USA, H	Platform programme description + deployment case studies	11.1 M de-identified patient records via Solutions Studio	Closed-loop Discover–Build–Deploy–Optimize architecture; pre-built EHR hooks; data-stays-local model federation	Operational continuous-validation, monitoring, and refinement infrastructure for deployed AI	V (infrastructure layer)
Duke Sepsis Watch [61]	USA, H	Implementation study (deployed since 2018)	32 M data points; 3 hospitals; ED workflow	Hourly DL sepsis scoring; iPad workflow; rapid-response escalation; SEP-1 bundle tracking	2x improvement in 3-h SEP-1 bundle compliance	V (single pathway)

continued on next page

https://doi.org/10.3390/healthcare14121612

Table S3 continued

Implementation	Setting	Design	Sample / population	Digital and analytical layer	Headline outcome	Type
Intermountain Care Process Models [62]	USA, H	Programme description + Heart Failure Pathway pilot	Pilot 5 hospitals (2015) → system-wide thereafter	Evidence-based CPMs in iCentra; outcome data fed back into "lean learning loop"	Heart Failure mortality 7 % pilot vs. 19 % non-pilot; discharge-home 34 % vs. 19 %	V (organisational)
VHA Preventive Health Inventory + AI inventory [63–65]	USA, H	Programme evaluation + national AI inventory	National Veterans population	PHI care management; antimicrobial-resistance ML; SoKat Suicidal Ideation Engine; immunosuppressant decision support	Diabetes control +2.9 pp; BP control +4.0 pp; equitable delivery across racial/ethnic groups	IV with V loops
Sheba ARC AI Center / Project K / AISAP POCUS [66,67]	Israel, H	Programme description + enterprise-wide deployment study	Hospital-wide internal medicine; ED triage pilot	Centralised AI Center for lifecycle and validation; AI ED triage; enterprise POCUS AI; AI Health Innovation Academy	2.5x ROI within one year; 30 % of POCUS cases changed clinical management	IV with V loops
FLORENCE Nordic federated colorectal-cancer AI [68,69]	Denmark–Norway, H	Federated registry-based development + national implementation	18 , 403 colorectal-cancer patients	OMOP-CDM federated learning across DCCG and Norwegian Cancer Registry; perioperative AI risk model	Reduction in serious perioperative complications vs. standard care	IV / V
UAE Riayati / Malaffi / Nabidh integration [70]	UAE, H	National HIE programme + integration milestone	2.7 B records; 11.6 M patients; 3 , 850 facilities; 90000 providers	National HIE federation across federal and emirate platforms; cross-emirate record access	Care-coordination, error-reduction and decision-support gains under integrated access	III/IV
Singapore HealthHub / NEHR + AI initiative [71]	Singapore, H	National programme + 2024 MOH AI fund	National public-healthcare population	NEHR; HealthHub AI multilingual chatbot; AimSG imaging platform; gen-AI documentation	SGD 200 M five-year MOH AI fund; progressive 2025 rollout across public-healthcare institutions	IV

continued on next page

https://doi.org/10.3390/healthcare14121612

Table S3 continued

Implementation	Setting	Design	Sample / population	Digital and analytical layer	Headline outcome	Type
Argentina FarmaTe-Cuida hypertension mHealth [72]	Argentina, UM	Implementation evaluation, 2020–2022	33 PHCs (23 adopting); General Pueyrredón, Buenos Aires	Community-pharmacy + PHC integration via mHealth	Implementation feasibility under pandemic conditions; uneven adoption documented	II/III
Colombia integrated multimorbidity model [73]	Colombia, UM	World Bank policy review of national framework	National multimorbidity population	PHC-anchored risk identification, stratification and care circuits; bio-psycho-social model	Programmatic restructuring around PHC; implementation early-stage	II/III
Ethiopia Data-Informed Platform for Health (DIPH) [74,75]	Ethiopia, LM	Cluster-randomised study, 24 districts	24 districts; subnational health-management cohort	DIPH data-driven decision-making at district level; DHIS2 backbone	+77 % regularity of monthly performance reviews; +48 % data-based feedback	II/III
Philippines UHC Act implementation [76–78]	Philippines, LM	Reform reflection + facility readiness assessment	National PHC network; Vietnam–Philippines facility audit	Inter-municipal cooperation; primary-care networks; digital health under UHC Act; local health-officer-led integration	Reform progress documented; persistent fragmentation and uneven primary-care readiness	II / III
COMFORTage Integrated Care Model Library / VHP [79,80]	MC, EU, H	Project-deliverable architecture + pilot design	Cross-country ageing-care pilots	Workflow of AI agents across ICM lifecycle; multimodal fusion; automated model selection; continuous learning; XAI; FHIR + blockchain audit	Trajectory-aware risk and equitable model performance under design and pilot	IV with V design intent

https://doi.org/10.3390/healthcare14121612

S4. Supplementary Policy Frameworks

Beyond the three anchor frameworks referenced in the Introduction (WHO IPCHS, UN Decade of Healthy Ageing, and the European Health Data Space), additional policy instruments cited in the review include the EU Green Paper on Ageing and the European Care Strategy [81,82]; the U.S. 21st Century Cures Act [83]; WHO's ICOPE guidance and regional integrated-care standards [84,85]; and national or provincial tools such as Canada's people-centred standards and Asia–Pacific digital-health strategies [86]. Together these instruments frame the legal, ethical, and organisational scaffolding on which Type IV and Type V deployments depend.

References

1. Tricco, A.C.; Lillie, E.; Zarin, W.; O'Brien, K.K.; Colquhoun, H.; Levac, D.; Moher, D.; Peters, M.D.J.; Horsley, T.; Weeks, L.; et al. PRISMA extension for scoping reviews (PRISMA-ScR): checklist and explanation. *Annals of Internal Medicine* **2018**, *169*, 467–473. https://doi.org/10.7326/M18-0850.
2. Arksey, H.; O'Malley, L. Scoping studies: towards a methodological framework. *International Journal of Social Research Methodology* **2005**, *8*, 19–32. https://doi.org/10.1080/1364557032000119616.
3. Levac, D.; Colquhoun, H.; O'Brien, K.K. Scoping studies: advancing the methodology. *Implementation Science* **2010**, *5*, 69. https://doi.org/10.1186/1748-5908-5-69.
4. Landis, J.R.; Koch, G.G. The measurement of observer agreement for categorical data. *Biometrics* **1977**, *33*, 159–174. https://doi.org/10.2307/2529310.
5. Harrison, S.R.; Jordan, A.M. Chronic disease care integration into primary care services in sub-Saharan Africa: a 'best fit' framework synthesis and new conceptual model. *Fam Med Community Health* **2022**, *10*, e001703. https://doi.org/10.1136/fmch-2022-001703.
6. Hwang, M.; Zheng, Y.; Cho, Y.; Jiang, Y. AI Applications for Chronic Condition Self-Management: Scoping Review. *J Med Internet Res* **2025**, *27*, e59632. https://doi.org/10.2196/59632.
7. Waring, J.; Lindvall, C.; Umeton, R. Automated machine learning: Review of the state-of-the-art and opportunities for healthcare. *Artificial Intelligence in Medicine* **2020**, *104*, 101822. https://doi.org/10.1016/j.artmed.2020.101822.
8. Denecke, K.; May, R.; Rivera-Romero, O. Transformer Models in Healthcare: A Survey and Thematic Analysis of Potentials, Shortcomings and Risks. *J Med Syst* **2024**, *48*, 23. https://doi.org/10.1007/s10916-024-02043-5.
9. Renc, P.; Jia, Y.; Samir, A.E.; Was, J.; Li, Q.; Bates, D.W.; Sitek, A. Zero Shot Health Trajectory Prediction Using Transformer, 2024. Pages: 2024.02.29.24303512, https://doi.org/10.1101/2024.02.29.24303512.
10. Sun, M.; Yang, X.; Niu, J.; Gu, Y.; Wang, C.; Zhang, W. A cross-modal clinical prediction system for intensive care unit patient outcome. *Knowledge-Based Systems* **2024**, *283*, 111160. https://doi.org/10.1016/j.knosys.2023.111160.
11. Kale, M.; Wankhede, N.; Pawar, R.; Ballal, S.; Kumawat, R.; Goswami, M.; Khalid, M.; Taksande, B.; Upaganlawar, A.; Umekar, M.; et al. AI-driven innovations in Alzheimer's disease: Integrating early diagnosis, personalized treatment, and prognostic modelling. *Ageing Research Reviews* **2024**, *101*, 102497. https://doi.org/10.1016/j.arr.2024.102497.
12. Kim, S.Y. Personalized Explanations for Early Diagnosis of Alzheimer's Disease Using Explainable Graph Neural Networks with Population Graphs. *Bioengineering* **2023**, *10*, 701. Number: 6 Publisher: Multidisciplinary Digital Publishing Institute, https://doi.org/10.3390/bioengineering10060701.
13. Sun, C.; Huang, S.; Pompili, D. LLM-based Multi-Agent Reinforcement Learning: Current and Future Directions, 2024. arXiv:2405.11106 [cs], https://doi.org/10.48550/arXiv.2405.11106.
14. Baltrušaitis, T.; Ahuja, C.; Morency, L.P. Multimodal Machine Learning: A Survey and Taxonomy, 2017. arXiv:1705.09406 [cs], https://doi.org/10.48550/arXiv.1705.09406.
15. Rieke, N.; Hancox, J.; Li, W.; Milletarì, F.; Roth, H.R.; Albarqouni, S.; Bakas, S.; Galtier, M.N.; Landman, B.A.; Maier-Hein, K.; et al. The future of digital health with federated learning. *NPJ Digit Med* **2020**, *3*, 119. https://doi.org/10.1038/s41746-020-00323-1.
16. Saberi, M.A.; Mcheick, H.; Adda, M. From Data Silos to Health Records Without Borders: A Systematic Survey on Patient-Centered Data Interoperability. *Information* **2025**, *16*, 106. Number: 2 Publisher: Multidisciplinary Digital Publishing Institute, https://doi.org/10.3390/info16020106.
17. Rafiei, A.; Moore, R.; Jahromi, S.; Hajati, F.; Kamaleswaran, R. Meta-learning in healthcare: A survey. *SN COMPUT. SCI.* **2024**, *5*, 791. arXiv:2308.02877 [cs], https://doi.org/10.1007/s42979-024-03166-9.
18. Baniecki, H.; Kretowicz, W.; Piątyszek, P.; Wiśniewski, J.; Biecek, P. dalex: Responsible Machine Learning with Interactive Explainability and Fairness in Python. *Journal of Machine Learning Research* **2021**, *22*, 1–7.

19. Salih, A.M.; Raisi-Estabragh, Z.; Galazzo, I.B.; Radeva, P.; Petersen, S.E.; Lekadir, K.; Menegaz, G. A Perspective on Explainable Artificial Intelligence Methods: SHAP and LIME. *Advanced Intelligent Systems* **2025**, *7*, 2400304. _eprint: https://onlinelibrary.wiley.com/doi/pdf/10.1002/aisy.202400304, https://doi.org/10.1002/aisy.202400304.
20. Whitehead, M. A typology of actions to tackle social inequalities in health. *J Epidemiol Community Health* **2007**, *61*, 473–478. https://doi.org/10.1136/jech.2005.037242.
21. Obermeyer, Z.; Powers, B.; Vogeli, C.; Mullainathan, S. Dissecting racial bias in an algorithm used to manage the health of populations. *Science* **2019**, *366*, 447–453. https://doi.org/10.1126/science.aax2342.
22. Gilfillan, R.J.; Tomcavage, J.; Rosenthal, M.B.; Davis, D.E.; Graham, J.; Roy, J.A.; Pierdon, S.B.; Bloom, F.J.; Graf, T.R.; Goldman, R.; et al. Value and the medical home: effects of transformed primary care. *The American Journal of Managed Care* **2010**, *16*, 607–614. PMID: 20712394.
23. Paulus, R.A. ProvenCare: Geisinger's model for care transformation through innovative clinical initiatives and value creation. *American Health & Drug Benefits* **2009**, *2*, 122–127. PMCID: PMC4106555.
24. Nguyen, D.; Lee, S.; Synghal, R.; Chan, L.; Justus, F.; Moromisato, M.; Shao, T.; Wang, C.; Kellogg, M.; Anwar, B.; et al. Digital transformation with clinical alerts and personalized care systems in an integrated value-based model: the Kaiser Permanente Intelligent Navigator. *npj Digital Medicine* **2025**. Reports KPIN deployment to 4.9M patients in Southern California Permanente Medical Group; clinical alert AUC 0.977; navigation 0.889, https://doi.org/10.1038/s41746-025-01838-1.
25. Kaiser Permanente Division of Research. Expanding care coordination in an integrated health system through causal machine learning. *Kaiser Permanente Division of Research Report* **2024**. Large randomised post-discharge care coordination evaluation, May–Dec 2022.
26. Embuldeniya, G.; McKellar, K.; Commisso, E.; Hall, R.; Wodchis, W.P. The Evolution of Ontario Health Teams: a developmental evaluation. *International Journal of Integrated Care* **2023**, *23*, 118. https://doi.org/10.5334/ijic.ICIC23118.
27. Embuldeniya, G.; Kirst, M.; Walker, K.; Wodchis, W.P. The generation of integration: the early experience of implementing bundled care in Ontario, Canada. *The Milbank Quarterly* **2018**, *96*, 782–813. https://doi.org/10.1111/1468-0009.12357.
28. Schubert, I.; Stelzer, D.; Siegel, A.; Köster, I.; Mehl, C.; Ihle, P.; Günster, C.; Dröge, P.; Klöss, A.; Farin-Glattacker, E.; et al. Ten-year evaluation of the population-based integrated health care system "Gesundes Kinzigtal": the INTEGRAL study. *Deutsches Ärzteblatt International* **2021**, *118*, 465–472. https://doi.org/10.3238/arztebl.m2021.0163.
29. Schubert, I.; Siegel, A.; Graf, E.; Farin-Glattacker, E.; Ihle, P.; Köster, I.; Stelzer, D.; Mehl, C.; Schmitz, J.; Dröge, P.; et al. Study protocol for a quasi-experimental claims-based study evaluating 10-year results of the population-based integrated healthcare model "Gesundes Kinzigtal": the INTEGRAL study. *BMJ Open* **2019**, *9*, e025945. https://doi.org/10.1136/bmjopen-2018-025945.
30. Commonwealth Fund. France. International Health Care System Profiles, The Commonwealth Fund, 2025. Available online: https://www.commonwealthfund.org/international-health-policy-center/countries/france (accessed on 2 June 2026).
31. NHS England. Federated Data Platform: building a connected NHS for the future. https://www.england.nhs.uk/digitaltechnology/digital-transformation-services/federated-data-platform/, 2024. Accessed: 2026-04-15.
32. Aula, V. Institutions, infrastructures, and data friction—reforming secondary use of health data in Finland. *Big Data & Society* **2019**, *6*, 2053951719875980. https://doi.org/10.1177/2053951719875980.
33. Nandram, S.S.; Koster, N. Organizational innovation and integrated care: lessons from Buurtzorg. *Journal of Integrated Care* **2014**, *22*, 174–184. https://doi.org/10.1108/JICA-06-2014-0024.
34. Cascini, F.; Gentili, A.; Melnyk, A.; Beccia, F.; Causio, F.A.; Solimene, V.; Battilomo, S.; Paone, S.; Borghini, A.; Bartolo, M.; et al. A new digital model for the Italian Integrated Home Care: strengths, barriers, and future implications. *Frontiers in Public Health* **2023**, *11*, 1292442. https://doi.org/10.3389/fpubh.2023.1292442.
35. Sano, J.; Hirazawa, Y.; Komamura, K.; Okamoto, S. An overview of systems for providing integrated and comprehensive care for older people in Japan. *Archives of Public Health* **2023**, *81*, 179. https://doi.org/10.1186/s13690-023-01076-5.
36. Otaga, M.; Yamaguchi, K.; Moriyama, Y.; Kakinuma, T. Changes in management initiatives in the long-term care insurance system by municipalities in Japan: toward the promotion of a community-based integrated care system. *International Journal of Integrated Care* **2023**, *23*, 178. https://doi.org/10.5334/ijic.APIC3178.
37. Cha, E.S.; Lee, Y.M.; Lee, S.M. Development and effectiveness of an AI chatbot–based mobile cognitive screening and customized training application for preventing dementia: older adults living in rural areas of South Korea. *Korean Journal of Adult Nursing* **2024**, *36*, 1–12. Pre-post study with 123 rural older adults.
38. Kim, D.J.; Lee, Y.S.; Jeon, E.R.; Kim, K.J. Present and future of AI-IoT-based healthcare services for senior citizens in local communities: a review of South Korean government digital-healthcare initiatives. *Healthcare* **2024**, *12*, 281. https://doi.org/10.3390/healthcare12020281.
39. Ga, H. The Korean long-term care system: 2024 update. *Annals of Geriatric Medicine and Research* **2024**, *28*, 235–237. https://doi.org/10.4235/agmr.24.0132.

40. Deng, H.; Wu, M.; Yang, Z.; Zhao, Q.; Zhu, L.; Dai, H.; Wu, J.; Liao, X.; Zhang, Y. Construction of an innovative model of chronic disease management in compact county medical alliance with treatment-prevention integration. *Chinese General Practice* **2023**, *26*, 2720–2725. Accessed: 2026-06-02.
41. Ding, S.; Zhou, Y. County medical community, medical insurance package payment, and hierarchical diagnosis and treatment: empirical analysis of the impact of the pilot project of compact county medical communities in Sichuan Province. *PLOS ONE* **2024**, *19*, e0297340. https://doi.org/10.1371/journal.pone.0297340.
42. Sum, G.; Sim, S.Y.H.; Chay, J.; Ho, S.H.; Ginting, M.L.; Lim, Z.Z.B.; Yoong, J.; Wong, C.H. An Integrated Patient-Centred Medical Home (PCMH) Care Model Reduces Prospective Healthcare Utilisation for Community-Dwelling Older Adults with Complex Needs: A Matched Observational Study in Singapore. *International Journal of Environmental Research and Public Health* **2023**, *20*, 6848. https://doi.org/10.3390/ijerph20196848.
43. Ladds, E.; Ivey, M.; Gadsby, K.; Preest, E.; Samuels, F.; Bradley, V. Continuity within a community integrated palliative care model and the influence of remote and digital approaches to care: a qualitative interview study. *BJGP Open* **2025**, *9*, BJGPO.2024.0126. https://doi.org/10.3399/BJGPO.2024.0126.
44. Li, M.; Chu, M.; Shen, Y.; Zhang, S.; Yin, X.; Yang, S.; Lip, G.Y.; Chen, M.; MIRACLE-AF Trial Investigators. A Novel Model of Integrated Care of Older Patients With Atrial Fibrillation in Rural China. *JACC Asia* **2024**, *4*, 764–773. https://doi.org/10.1016/j.jacasi.2024.07.006.
45. Guo, R.; Zhang, J.; Yang, F.; Wu, Y. Efficacy of an Intelligent and Integrated Older Adult Care Model on Quality of Life Among Home-Dwelling Older Adults: Randomized Controlled Trial. *Journal of Medical Internet Research* **2025**, *27*, e67950. https://doi.org/10.2196/67950.
46. Macinko, J.; Harris, M.J. Brazil's Family Health Strategy — delivering community-based primary care in a universal health system. *New England Journal of Medicine* **2015**, *372*, 2177–2181. https://doi.org/10.1056/NEJMp1501140.
47. Cavalcante, D.F.A.; Brizon, V.S.C.; Probst, L.F.; Meneghim, M.d.C.; Pereira, A.C.; Ambrosano, G.M.B. Did the family health strategy have an impact on indicators of hospitalizations for stroke and heart failure? *Health Policy and Planning* **2021**, *36*, 821–827. https://doi.org/10.1093/heapol/czab031.
48. Borja-Aburto, V.H. La atención primaria en el Modelo de Atención a la Salud para el Bienestar en México. *Salud Pública de México* **2024**, *66*, 670–676. https://doi.org/10.21149/15755.
49. Ramonfaur, D.; Torres-Martínez, M. Effectuating worthy medical training without neglecting health services in Mexico. *The Lancet Regional Health – Americas* **2024**, *34*, 100739. https://doi.org/10.1016/j.lana.2024.100739.
50. Mora-García, C.A.; Pearson, A.A.; Prado, A.M. Maintaining essential health services during a pandemic: lessons from Costa Rica's COVID-19 response. *BMJ Global Health* **2024**, *8*, e014143. https://doi.org/10.1136/bmjgh-2023-014143.
51. Rubuga, F.K.; Absolomon, G.; Uhawenimana, T.C.; Nsaba-Uwera, Y.D.; Muhire, J.; Hagenimana, J.D.; Nyabyenda, E.C.; Irakiza, P.; Semakula, M.; Remera, E.; et al. Telemedicine implementation and healthcare utilization in Rwanda: interrupted time series of babyl digital-health services from 2015 to 2024. *BMC Primary Care* **2026**, *27*, 49. 3.9M consultations; 450/510 facilities; task-shifting outcomes, https://doi.org/10.1186/s12875-026-03179-8.
52. Musange Furere, S.; Absolomon, G.; Umutoni, N.; Matutina Umuhoza, S.; K Rubuga, F.; Nsaba-Uwera, Y.D.; Singa Muhoza, P.; Hitimana, R.; Condo, J.; Humuza, J. Digital primary health in Rwanda: qualitative study of user experiences and implementation lessons from babyl's telemedicine platform. *Journal of Medical Internet Research* **2026**, *28*, e84832. https://doi.org/10.2196/84832.
53. Alshehri, A.A.; Abduljawad, A.A. Impact of the Saudi Health Sector Transformation Program (SHSTP): a mixed-methods evaluation of patient-centered care and digital-health adoption. *Healthcare* **2025**, *13*, 2968. https://doi.org/10.3390/healthcare13222968.
54. Mani, Z.A.; Goniewicz, K. Transforming healthcare in Saudi Arabia: a comprehensive evaluation of Vision 2030's impact. *Sustainability* **2024**, *16*, 3277. https://doi.org/10.3390/su16083277.
55. Lahariya, C. 'Ayushman Bharat' programme and Universal Health Coverage in India. *Indian Pediatrics* **2018**, *55*, 495–506. https://doi.org/10.1007/s13312-018-1341-1.
56. Prinja, S.; Dixit, J.; Nimesh, R.; Garg, B.; Khurana, R.; Paliwal, A. Impact of health benefit package policy interventions on service utilisation under government-funded health insurance in Punjab, India: analysis of Ayushman Bharat Pradhan Mantri Jan Arogya Yojana (PM-JAY). *The Lancet Regional Health – Southeast Asia* **2024**, *28*, 100462. https://doi.org/10.1016/j.lansea.2024.100462.
57. Tangcharoensathien, V.; Witthayapipopsakul, W.; Panichkriangkrai, W.; Patcharanarumol, W.; Mills, A. Health systems development in Thailand: a solid platform for successful implementation of universal health coverage. *The Lancet* **2018**, *391*, 1205–1223. https://doi.org/10.1016/S0140-6736(18)30198-3.
58. Australian Digital Health Agency. Annual Report 2023–24. Australian Digital Health Agency, 2024. 26 percent rise in My Health Record consumer interactions; 90 percent of Australians have a record.

59. Australian Digital Health Agency. National Healthcare Interoperability Plan: Q2 October–December 2024 progress report, 2024.
60. Mayo Clinic Platform. Deployment is King: continuous learning and optimization in the Mayo Clinic Platform. Mayo Clinic Platform, 2024. Discover-Build-Deploy-Optimize closed-loop learning architecture; 11.1M de-identified patient records via Solutions Studio.
61. Sendak, M.P.; Ratliff, W.; Sarro, D.; Alderton, E.; Futoma, J.; Gao, M.; Nichols, M.; Revoir, M.; Yashar, F.; Miller, C.; et al. Real-world integration of a sepsis deep learning technology into routine clinical care: implementation study. *JMIR Medical Informatics* **2020**, *8*, e15182. Duke Sepsis Watch deployment study, https://doi.org/10.2196/15182.
62. Intermountain Healthcare. Four steps to a learning healthcare system. Intermountain Healthcare, 2016. Care Process Models and the lean learning loop; Heart Failure Pathway mortality 7% vs. 19% in pilot.
63. Wheat, C.L.; Reddy, A.; Shirley, S.E.; Gray, K.E.; Stockdale, S.E.; Nelson, K.M.; Wong, E.S. Population-level health intervention and primary care quality for Veterans: the VHA Preventive Health Inventory. *JAMA Network Open* **2025**, *8*, e2544378. Diabetes control +2.9 pp; BP control +4.0 pp under PHI care management, https://doi.org/10.1001/jamanetworkopen.2025.44378.
64. U.S. Department of Veterans Affairs. VA AI Inventory 2024. Department of Veterans Affairs Office of AI Implementation, 2024. Antimicrobial resistance detection; SoKat Suicidal Ideation Engine; medication-optimisation AI.
65. Marcotte, L.M.; Wheat, C.L.; Rao, M.; Wong, E.S.; Hebert, P.; Nelson, K.; Rojas, J.; Gunnink, E.J.; Reddy, A. Evaluating equity in a national virtual care management intervention: delivery and outcomes by race/ethnicity among Veterans with hypertension and diabetes. *Health Services Research* **2024**, *59*, e14352. https://doi.org/10.1111/1475-6773.14352.
66. Akselrod-Ballin, A.; Zimlichman, E.; et al. Building the future of AI-driven hospitals: the Sheba ARC AI Center, Project K emergency-room triage, and the AI Health Innovation Academy. *Sheba Global Innovation Reports* **2025**.
67. Topilsky, Y.; Sherez, J.; et al. Enterprise-wide implementation of AI-powered point-of-care ultrasound at Sheba Medical Center: a 2.5x ROI case study. *AISAP Case Studies* **2024**.
68. Mosegaard, S.B.; Buhl, S.; Christensen, J.F.; et al. FLORENCE: federated learning on OMOP-modelled Nordic cancer-registry data for colorectal-cancer decision support. *NIPH Project Reports* **2025**. 2022–2025; Denmark–Norway federated colorectal-cancer AI under OMOP-CDM.
69. Rosen, A.W.; Ose, I.; Gögenur, M.; Andersen, L.P.K.; Bojesen, R.D.; Vogelsang, R.P.; Rose, M.H.; Steenfos, P.W.; Hansen, L.B.; Spuur, H.S.; et al. Clinical implementation of an AI-based prediction model for decision support for patients undergoing colorectal cancer surgery. *Nature Medicine* **2025**, *31*, 3737–3748. https://doi.org/10.1038/s41591-025-03942-x.
70. Malaffi. UAE health authorities announce successful integration between Riayati, Malaffi, and Nabidh. Malaffi (Abu Dhabi Health Data Services), 2023. 2.7B medical records; 11.6M patients; 3,850 facilities; 90000 providers.
71. Synapxe. HealthHub, National Electronic Health Record, and the MOH Health Innovation Fund AI initiative. Synapxe and Singapore Ministry of Health, 2024. SGD 200M five-year MOH AI fund; HealthHub AI chatbot; AimSG imaging platform.
72. Esandi, M.E.; Ortiz, Z.; Bernabei, V.; Villalba, N.B.; Liggio, S.; Della Maggiora, M.; García, N.A.; Bruzzone, A.; Blanco, G.; Prieto Merino, D.; et al. Evaluating the implementation of a hypertension program based on mHealth and community-pharmacies integration to primary-care centres at a municipality level in Argentina during the COVID-19 pandemic. *Frontiers in Health Services* **2024**, *4*, 1263331. FarmaTeCuida programme; 33 PHCs; 23 adopting Oct 2020–Mar 2022, https://doi.org/10.3389/frhs.2024.1263331.
73. World Bank. Comprehensive healthcare management model for people with multimorbidity in Colombia: a primary-health-care framework. *World Bank Knowledge Brief* **2024**.
74. Avan, B.I.; Dubale, M.; Taye, G.; Marchant, T.; Persson, L.A.; Schellenberg, J. Data-driven decision-making for district health management: a cluster-randomised study in 24 districts of Ethiopia. *BMJ Global Health* **2024**, *9*, e014140. DIPH; +77% regularity of monthly performance reviews; +48% data-based feedback, https://doi.org/10.1136/bmjgh-2023-014140.
75. Simbini, T.; Adimado, E.; Adjorlolo, S.; Guerrero-Torres, L.; Srinivas, P.; Zizhou, S.; Zerfu, T. Digital health interventions in strengthening primary healthcare systems in Sub-Saharan Africa: insights from Ethiopia, Ghana, and Zimbabwe. *PLOS Digital Health* **2026**, *5*, e0000863. https://doi.org/10.1371/journal.pdig.0000863.
76. Co, P.A.; Vîlcu, I.; De Guzman, D.; Banzon, E. Staying the course: reflections on the progress and challenges of the UHC Law in the Philippines. *Health Systems and Reform* **2024**, *10*, 2397829. https://doi.org/10.1080/23288604.2024.2397829.
77. de Claro, V.; Lava, J.B.; Bondoc, C.; Stan, L. The role of local health officers in advancing public health and primary care integration: lessons from the ongoing Universal Health Coverage reforms in the Philippines. *BMJ Global Health* **2024**, *9*, e014118. https://doi.org/10.1136/bmjgh-2023-014118.
78. Fernandez, M.L.; Nguyen, H.; Nguyen, D.; Holt, B.; Doan, D.; Gaspar, M.; Hamoy, G.; Mendoza, J.; Mercado, T.B.; Cabauatan, D.J.; et al. Healthcare system readiness to manage viral hepatitis in Viet Nam and the Philippines: results of a brief health-facility assessment. *BMC Health Services Research* **2026**, *26*, 88. https://doi.org/10.1186/s12913-026-14088-y.
79. Manias, G.; Likothanassis, S.; Alexakis, E.; Antoniades, A.; Marra, C.; Giuffrè, G.M.; Charalambous, E.; Tsolis, D.; Tsirogiannis, G.; Koutsomitropoulos, D.; et al. Establishing a Digitally Enabled Healthcare Framework for Enhanced

Prevention, Risk Identification, and Relief for Dementia and Frailty. *J. Dement. Alzheimer's Dis.* **2025**, *2*, 30. https://doi.org/10.3390/jdad2030030.

80. Tsitiridis, A.; Alexakis, M.; Tsolis, D.; Lykothanasi, K.; Tsoukalos, D.; Koutsomitropoulos, D.; Giannaros, A.; Manias, G. D3.7 Integrated AI-based Care Model Library I. Project deliverable, COMFORTage Consortium, Horizon Europe Grant Agreement No. 101137301, 2025. Public deliverable, Work Package 3, Tasks T3.5–T3.6.
81. Commission, E. Green paper on ageing - Publications Office of the EU.
82. Caracciolo di Torella, E. Re-thinking care after the pandemic: a European Care Strategy for Caregivers and Care Receivers. *ERA Forum* **2023**, *24*, 55–67. https://doi.org/10.1007/s12027-023-00744-x.
83. Office of the National Coordinator for Health Information Technology (ONC), D.o.H.a.H.S.H. 21st Century Cures Act: Interoperability, Information Blocking, and the ONC Health IT Certification Program. Technical report, U.S. HHS, 2020.
84. (WHO), W.H.O. Integrated care for older people: guidelines on community-level interventions to manage declines in intrinsic capacity. Technical report, WHO, Geneva, 2017.
85. (WHO), W.H.O. WHO Western Pacific Region Integrated Care Report 2022. Technical report, WHO in the Western Pacific Region, 2022.
86. Sullivan-Taylor, P.; Suter, E.; Laxton, S.; Oelke, N.D.; Park, E. Integrated People-Centred Care in Canada – Policies, Standards, and Implementation Tools to Improve Outcomes. *Int J Integr Care*, *22*, 8. https://doi.org/10.5334/ijic.5943.
